# An emergent biofilm program from inactivation of *Candida albicans* master regulators Efg1 and Ndt80

**DOI:** 10.1371/journal.ppat.1014469

**Published:** 2026-07-20

**Authors:** Eunsoo Do, Robert Zarnowski, David R. Andes, Aaron P. Mitchell

**Affiliations:** 1 Department of Microbiology, University of Georgia, Athens, GeorgiaUnited States of America; 2 Department of Medical Microbiology and Immunology, University of Wisconsin, Madison, Wisconsin, United States of America; State Key Laboratory of Oral Diseases, CHINA

## Abstract

Biofilm formation by the fungus *Candida albicans* is a central virulence trait that enables colonization of implanted medical devices and mucosal surfaces. Biofilm formation reflects a complex regulatory network, and depends upon multiple master regulators that include transcription factors Efg1 and Ndt80. It is well established that *efg1*Δ/Δ and *ndt80*Δ/Δ single gene mutants are defective in biofilm formation. We report here that an *efg1*Δ/Δ *ndt80*Δ/Δ double mutant of reference strain SC5314 is able to form a robust biofilm in vitro and in vivo. We refer to the *efg1*Δ/Δ *ndt80*Δ/Δ biofilm as an emergent biofilm because this phenotype could not have been predicted from the phenotypes of *efg1*Δ/Δ or *ndt80*Δ/Δ single gene mutants. In four additional strain backgrounds, *efg1*Δ/Δ *ndt80*Δ/Δ mutants do not form biofilms, but in all strain backgrounds the *efg1*Δ/Δ *ndt80*Δ/Δ mutants can form filamentous cells, which are components of biofilms. Emergent biofilm formation is especially pronounced in YPD + FBS medium at 30°C, and RNA-seq under those conditions reveals altered expression in the *efg1*Δ/Δ *ndt80*Δ/Δ double mutant of biofilm-related genes: upregulation of *BCR1, UME6,* and *HGC1*, and downregulation of *ALS3, BRG1,* and *HWP1.* These gene expression changes suggest that the emergent biofilm program is partially distinct from the conventional biofilm program. This inference is supported by functional analysis: emergent biofilm formation is independent of Brg1, Rob1, Tec1, and Wor3, all of which have positive roles in conventional biofilm formation. Emergent biofilm formation depends upon the hyphal cyclin Hgc1, the biofilm transcription factors Bcr1 and Ume6, and the Bcr1/Ume6-activated adhesin gene *FLO9*. The seemingly simple emergent biofilm program may represent a primordial surface colonization strategy.

## Author summary

Biofilm formation by pathogenic microbes enables colonization of implanted devices and, ultimately, infection. In the fungal pathogen *Candida albicans*, several master transcriptional regulators are required for biofilm formation and the associated gene expression program. Remarkably, while loss of either of the master regulators Efg1 or Ndt80 blocks biofilm formation, we find that loss of both Efg1 and Ndt80 together activates an emergent biofilm program. Many genes required for the conventional biofilm program are dispensable for the emergent biofilm program. We suggest that the emergent program represents a primordial surface colonization strategy that may have been the starting point for evolution of the conventional biofilm program.

## Introduction

Biofilm formation enables microbes to colonize surfaces [1], with serious consequences for human health. The biofilm state allows pathogens to persist on implanted medical devices and host tissues, creating a nidus for infection [1,2]. It is estimated that over 80% of human microbial infections are biofilm-associated, with an annual global cost in US dollars of ~$400 billion in 2019 [3].

This study we present here examines biofilm formation by *Candida albicans* [2], a fungal commensal and pathogen. Biofilm-related infections by *C. albicans* and other *Candida* species occur at diverse anatomical sites and cause an array of clinical diseases [2,4–6]. Current limits in therapeutic options may be overcome by an improved understanding of the genetic determinants of surface colonization and how they are coordinated.

Complexity seems to be an understatement when we describe *C. albicans* biofilm formation. It depends upon over 150 genes based on mutant phenotypes [7], and 1000 genes or more based on differential expression [8–10]. It is controlled by six master regulators, which are transcription factors required for biofilm formation in vitro and in vivo in the SC5314 reference strain background [8]. Analysis of directly bound target genes indicates that most master regulators function as both activators and repressors [8,11,12], though most functional biofilm-relevant targets analyzed to date are activated by the master regulators. Some master regulators, such as Efg1, form phase-separated condensates with one another [13,14]. Strong evidence indicates that these phase-separated condensates are necessary for activation of biofilm formation and the related process of filamentation [14].

Our study focuses on two master regulators, Efg1 and Ndt80. Efg1 is a basic helix-loop-helix transcription factor, and is among the most well-studied *C. albicans* gene products [15]. It is required for biofilm formation at 37°C under every condition examined [12,15,16], and in 18 of 18 genetic backgrounds tested [17–19]. It is also required for filamentation, a cell morphology transition critical for biofilm formation [2]. Efg1 can function as both an activator and a repressor [8,11,12]. In fact, some direct targets are activated by Efg1 in reference strain SC5314 but repressed by Efg1 in other strains [11,19]. Ndt80 belongs to a large eukaryotic DNA-binding protein family. It is required in *C. albicans* for biofilm formation and filamentation, as well as normal azole drug sensitivity [8,20–22]. Ndt80 is required for biofilm formation in 6 of 6 genetic backgrounds tested [23]. Our interest in these two master regulators was spurred on recently when we discovered that they each interact with the biofilm and filamentation regulator Ume6 [24], and are required for Ume6 to bind to and activate several biofilm-related genes. The studies summarized above led us to expect that the loss of Efg1 and Ndt80 together would cause a severe biofilm defect.

## Results

### Emergent biofilm formation and filamentation

Mutations *efg1*Δ/Δ and *ndt80*Δ/Δ each cause defects in biofilm formation and filamentation in multiple *C. albicans* clinical isolate backgrounds. We anticipated that an *efg1*Δ/Δ *ndt80*Δ/Δ double mutant would be defective in biofilm formation and filamentation as well. This was not the case, as revealed in two kinds of assay outcomes: one in which both *efg1*Δ/Δ and *ndt80*Δ/Δ single mutants are severely defective, and a second in which an *efg1*Δ/Δ single mutant is severely defective but an *ndt80*Δ/Δ single mutant is partially defective.

**Fig 1:**
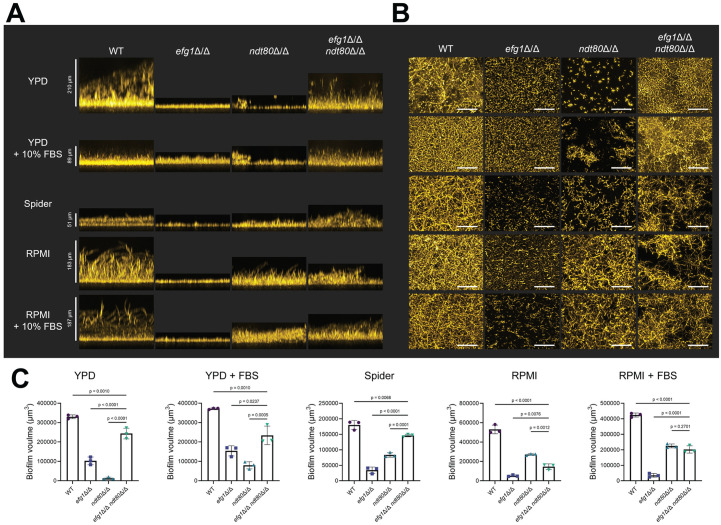
Emergent biofilm formation. **(A)** and **(B)** For biofilm formation, cells were grown in the indicated medium at 37°C for 24 hours and stained with Calcofluor-White. The images represent three biological replicates. Side- (A) and apical-view (B) images are shown. Scale bars in the apical-view images indicate 100 microns. **(C)** The bar graphs depict biofilm volume measurements. Statistical significance was determined using one-way ANOVA (Tukey’s multiple comparisons test). Values are the mean ±  s.d. of three biological replicates.

We constructed an *efg1*Δ/Δ *ndt80*Δ/Δ double mutant in the SC5314 reference strain background and assessed biofilm formation under multiple in vitro conditions. Cells of the wild type, each single mutant, and the double mutant were grown in a 96-well biofilm format for 24 hours at 37°C in media that included YPD, YPD + FBS, Spider, RPMI, and RPMI+FBS. The wild type formed biofilms in all media, as indicated by structural depth of ~50–200 µm (Fig 1A), abundant filamentation (Fig 1B), and a biofilm volume of ~2-5x10^5^ µm^3^ (Fig 1C). The *efg1*Δ/Δ and *ndt80*Δ/Δ single mutants were defective, as indicated by reductions in those properties (Fig 1A–1C); the magnitude of the defect varied with the growth medium. The *efg1*Δ/Δ *ndt80*Δ/Δ double mutant produced biofilms more efficiently than either component single mutant in YPD, YPD + FBS, and Spider (Fig 1A–1C), with depth and volume approaching the wild type. The double mutant was partially defective in RPMI and RPMI+FBS (Fig 1A–1C), yielding biofilms that were comparable to the *ndt80*Δ/Δ single mutant. Under all conditions, filamentous cells in *efg1*Δ/Δ *ndt80*Δ/Δ biofilms were evident in side views (Fig 1A), apical views (Fig 1B), or both. We conclude that the *efg1*Δ/Δ *ndt80*Δ/Δ double mutant can produce biofilm under conditions in which each component single mutant is biofilm-defective. Formation of biofilm by the double mutant represents a synthetic genetic interaction [25]. Because biofilm formation by the double mutant could not have been predicted from the biofilm defects of either component single mutant, we refer to this *efg1*Δ/Δ *ndt80*Δ/Δ phenotype as emergent biofilm formation.

**Fig 2:**
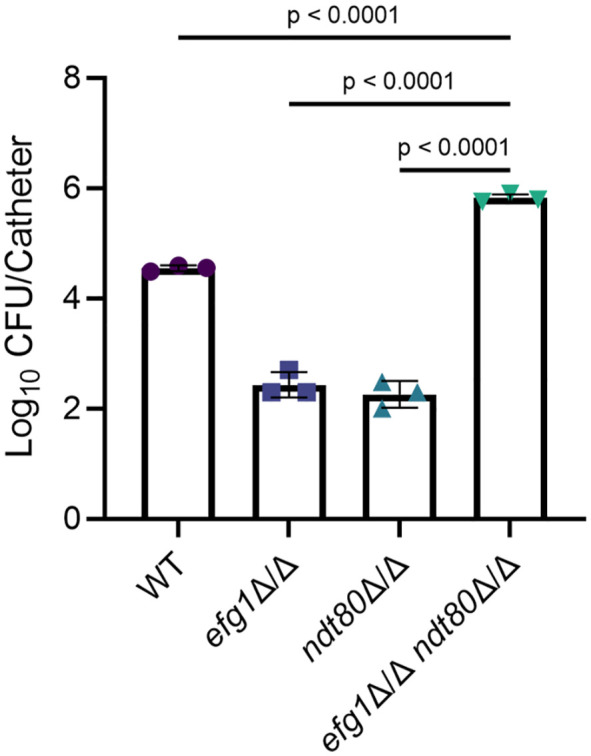
In vivo biofilm formation. SC5314 wild-type, *efg1*Δ/Δ, *ndt80*Δ/Δ, and *efg1*Δ/Δ *ndt80*Δ/Δ strains were tested for in vivo biofilm formation in a rat venous catheter infection model. *C. albicans* cell counts per catheter were determined at 48 hours post-infection. The graph presents three independent experiments. Statistical significance was determined using one-way ANOVA (Tukey’s multiple comparisons test). Values are the mean ±  s.d. of three biological replicates.

To see whether emergent biofilm formation occurs under infection conditions, we turned to a rat venous catheter biofilm model [26]. We assessed biofilm formation with triplicate samples of the wild type, the *efg1*Δ/Δ and *ndt80*Δ/Δ single mutants, and the *efg1*Δ/Δ *ndt80*Δ/Δ double mutant. Three catheterized animals were used to assay each strain (Fig 2). The wild-type strain yielded an average of ~10^4.6^ colony forming units (CFU) per catheter, indicative of effective colonization. The *efg1*Δ/Δ and *ndt80*Δ/Δ single mutants yielded ~10^2.5^ CFU per catheter, in keeping with the established biofilm defects of *efg1*Δ/Δ and *ndt80*Δ/Δ mutants in this model [8]. Remarkably, the *efg1*Δ/Δ *ndt80*Δ/Δ double mutant yielded ~10^5.8^ CFU per catheter, a colonization level well beyond that of either single mutant or the wild-type strain. These results show that emergent biofilm formation by the *efg1*Δ/Δ *ndt80*Δ/Δ double mutant can occur in the context of an in vivo infection model.

**Fig 3:**
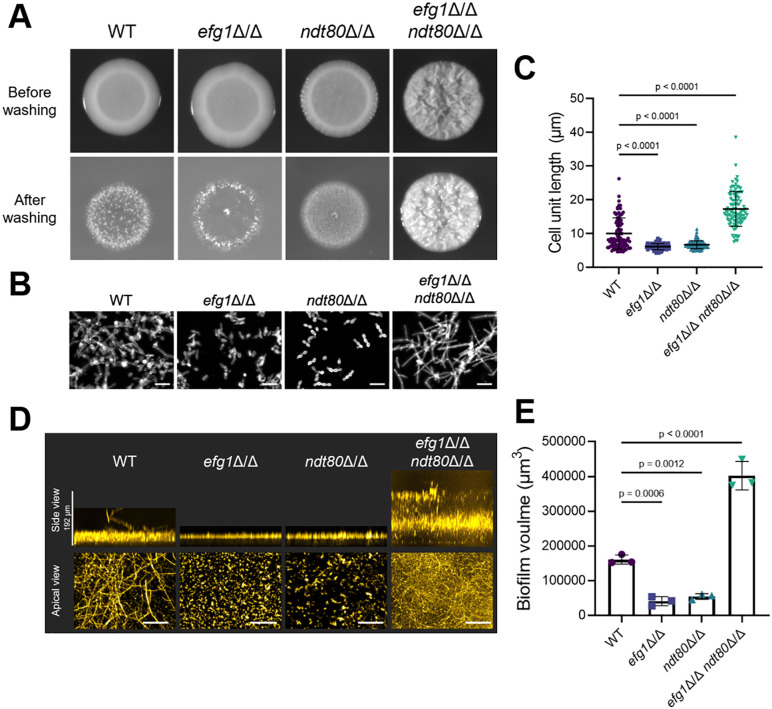
Emergent biofilm-associated phenotypes. **(A)** Strains were grown on YPD + FBS agar medium at 30°C for 3 days, then the plate was washed with water to test invasive growth. **(B)** For filamentation assays, cells were grown in YPD + FBS at 30°C for 4 hours and stained with Calcofluor-White. The white scale bar indicates 20 microns. **(C)** The scatter plot indicates cell unit length of each strain. Cell lengths were measured using a minimum of 100 cells from 3 different fields. Statistical significance was determined using one-way ANOVA (Tukey’s multiple comparisons test). The line and error bars indicate the mean ±  s.d. **(D)** For biofilm formation, cells were grown in YPD + FBS at 30°C for 24 hours and stained with Calcofluor-White. The images represent three biological replicates. Side-, apical- and navigation-view images are shown. Scale bars in the apical-view images indicate 100 microns. **(E)** The bar graph depicts biofilm volume measurements. Statistical significance was determined using one-way ANOVA (Tukey’s multiple comparisons test). Values are the mean ±  s.d. of three biological replicates.

For more detailed analysis, we developed conditions in which the emergent biofilm phenotype was robust. We had noticed that the *efg1*Δ/Δ *ndt80*Δ/Δ double mutant produced wrinkled colonies at 30°C, so we tested its phenotype in YPD + FBS medium at 30°C. Compared to the wild-type, *efg1*Δ/Δ, and *ndt80*Δ/Δ strains, the *efg1*Δ/Δ *ndt80*Δ/Δ double mutant produced highly wrinkled colonies on solid medium that were resistant to washing from the plate (Fig 3A). Liquid YPD + FBS was mildly inducing for filamentation of the wild type, and did not induce filamentation of the *efg1*Δ/Δ and *ndt80*Δ/Δ single mutants (Fig 3B, 3C). The *efg1*Δ/Δ *ndt80*Δ/Δ double mutant underwent filamentation to a greater extent than the wild type (Fig 3B, 3C). We refer to filamentation of the *efg1*Δ/Δ *ndt80*Δ/Δ double mutant under these conditions as emergent filamentation because it is distinct and opposite from the phenotype of either component single mutant. Emergent biofilm formation was evident as well under these conditions, as reflected in biofilm depth, filamentous cell content, and biofilm volume (Fig 3D, 3E). Complementation by integration of an *NDT80*-FLAG cassette into the *efg1*Δ/Δ *ndt80*Δ/Δ double mutant reverted its filamentation and biofilm phenotypes to that of the *efg1*Δ/Δ mutant (S1 Fig), indicating that the phenotype was not due to a secondary mutation. These results show that YPD + FBS medium at 30°C supports emergent filamentation and biofilm formation by the *efg1*Δ/Δ *ndt80*Δ/Δ double mutant.

**Fig 4:**
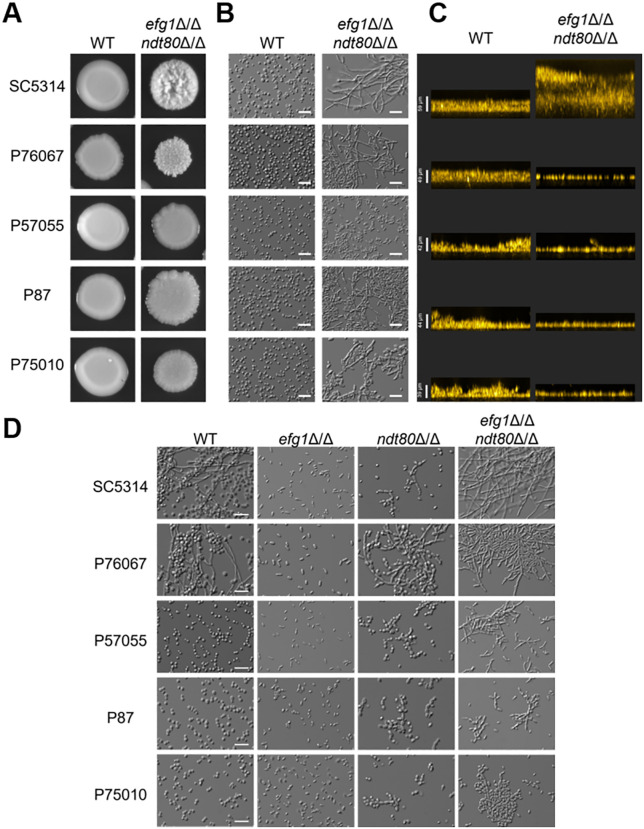
Strain dependence. **(A)** Strains were grown on YPD + 10% FBS agar medium at 30°C for 3 days. **(B)** Cells grown on YPD + 10% FBS agar medium at 30°C for 3 days were resuspended in PBS and imaged. Scale bars indicate 20 microns. **(C)** For biofilm formation, cells were grown in YPD + 10% FBS at 30°C for 24 hours and stained with Calcofluor-White. A side-view projection is shown. **(D)** Cells were grown in YPD + 10% FBS at 30°C for 24 hours under biofilm conditions and imaged. Scale bars indicate 20 microns.

We sought to determine whether emergent filamentation and biofilm formation were common features of *efg1*Δ/Δ *ndt80*Δ/Δ double mutants in multiple *C. albicans* strain backgrounds. We constructed *efg1*Δ/Δ *ndt80*Δ/Δ double mutants in a panel of clinical isolates used in previous genotype-phenotype analyses – P76067, P57055, P87, and P75010 – and examined colony morphology, filamentation, and biofilm formation in YPD + FBS at 30°C. A rough colony morphology was observed in double mutants of the P76067, P87, and P75010 backgrounds, though it was not as extreme as in the SC5314 background (Fig 4A). Filamentation of the double mutant was evident in these same three backgrounds (Fig 4B). Biofilm formation by the double mutant was apparent only in SC5314 under these conditions (Fig 4C). However, in the P76067, P57055, and P87 strain backgrounds, the double mutants produced filamentous cells under biofilm culture conditions (Fig 4D). We conclude that emergent biofilm formation is strain-limited, whereas altered colony morphology and emergent filamentation are common features of *efg1*Δ/Δ *ndt80*Δ/Δ double mutants in several clinical isolate backgrounds.

### Gene expression features of the emergent phenotype

**Fig 5:**
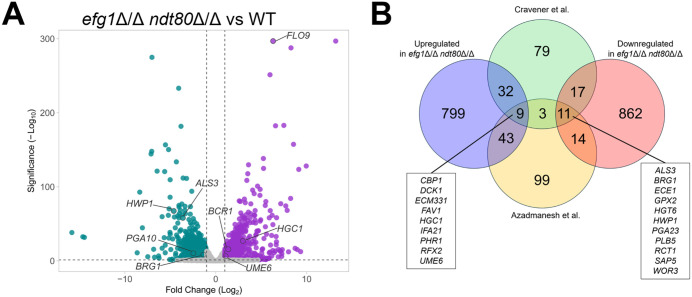
Gene expression impact of the *efg1*Δ/Δ *ndt80*Δ/Δ genotype. RNA-seq was conducted on the wild type and *efg1*Δ/Δ *ndt80*Δ/Δ double mutant from the SC5314 background. Strains were grown in YPD + FBS liquid medium at 30°C for 4 hours with shaking, a condition that supports emergent filamentation (see Fig 4B). **(A)** Volcano plots show differentially expressed genes in comparisons between *efg1*Δ/Δ *ndt80*Δ/Δ mutant and wild type. Each dot indicates a gene, and significant up- or down-regulation is reflected by a log2 fold-change of greater than 1 or less than -1, respectively, all with padj < 0.05. **(B)** Venn diagram depicts intersection of the upregulated and downregulated genes in the *efg1*Δ/Δ *ndt80*Δ/Δ mutant compared to the wild-type strain, as well as the Cravener et al. [17] and Azadmanesh et al. [27] hypha-associated gene sets. Common hypha-associated genes that are upregulated or downregulated are listed. RNA-seq analysis by Cravener et al. [17] was conducted on multiple different strains grown in RPMI+FBS medium at 37°C, and strains were compared to one another to identify gene expression features that correlated with filamentation ability. RNA-seq analysis by Azadmanesh et al. [27] was conducted on strain SC5314 in diverse filamentation-inducing liquid and solid media, including RPMI, Lee’s, Spider, or FBS, all at 37°C, and compared to cells grown in (filamentation noninducing) liquid YPD medium at 30°C.

To understand the transcriptional features of the emergent filamentation program, we performed RNA-seq on the wild type and *efg1*Δ/Δ *ndt80*Δ/Δ double mutant from the SC5314 background grown in YPD + FBS at 30°C for 4 hours with shaking. This condition supports emergent filamentation (Fig 4B). There was extensive remodeling of the transcriptome in the *efg1*Δ/Δ *ndt80*Δ/Δ mutant compared to the wild type, with 904 genes downregulated and 883 genes upregulated (Fig 5A, 5B, S1 Table).

The *efg1*Δ/Δ *ndt80*Δ/Δ double mutant transcriptome seemed to represent a non-canonical hyphal transcriptional profile. For example, the double mutant exhibited reduced expression, compared to the wild type, of several genes associated with filamentation and biofilm formation, including the cell wall adhesin genes *ALS3* and *HWP1*, and the biofilm regulator *BRG1* (Fig 5A, S1 Table). The double mutant exhibited increased expression of other genes associated with filamentation and biofilm formation, including hyphal cyclin gene *HGC1* and biofilm regulatory genes *BCR1* and *UME6* (Fig 5A, S1 Table). Hypha-associated gene sets defined by comparisons among diverse growth conditions by Azadmanesh et al. [27] or among diverse strains by Cravener et al. [17] were split among upregulated and downregulated gene sets in the *efg1*Δ/Δ *ndt80*Δ/Δ vs wild type comparison (Fig 5B). These observations suggest that emergent filamentation and biofilm formation by the *efg1*Δ/Δ *ndt80*Δ/Δ double mutant may reflect a regulatory program that overlaps only partially with that of the wild-type strain.

### Regulators of the emergent biofilm state

**Fig 6:**
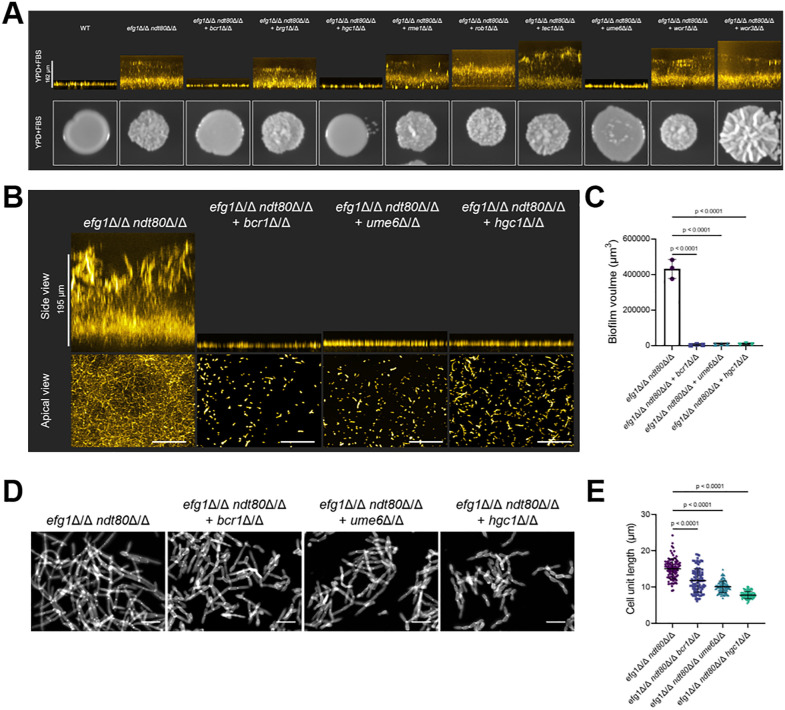
Regulation of emergent biofilm formation. **(A)** For biofilm formation, cells were grown in YPD + FBS at 30°C for 24 hours and stained with Calcofluor-White. Side-view projection images are shown. For colony morphology analysis, strains were grown on YPD + FBS agar medium at 30°C for 3 days. **(B)** For biofilm formation, cells were grown in YPD + FBS at 30°C for 24 hours and stained with Calcofluor-White. Side-view projection and apical view images are shown. Scale bars in the apical-view images indicate 100 microns. **(C)** The bar graph depicts biofilm volume measurements. Statistical significance was determined using one-way ANOVA (Tukey’s multiple comparisons test). Values are the mean ±  s.d. of three biological replicates. **(D)** For filamentation assay, cells were grown in YPD + FBS at 30°C for 4 hours and stained with Calcofluor-White. The white scale bar indicates 20 microns. **(E)** The scatter plot indicates cell unit length of each strain. Cell lengths were measured using a minimum of 100 cells from 3 different fields. The images represent three independent experiments. Statistical significance was determined using one-way ANOVA (Tukey’s multiple comparisons test). The line and error bars indicate the mean ±  s.d.

To identify regulators of this emergent state, we conducted a targeted deletion mutant screen. Individual biofilm transcriptional regulators were deleted in the *efg1*Δ/Δ *ndt80*Δ/Δ strain, including *BCR1*, *BRG1*, *ROB1*, *TEC1*, *UME6*, *WOR1*, and *WOR3*. We also deleted the hyphal cyclin gene *HGC1*. We then assayed colony morphology and biofilm formation in YPD + FBS medium at 30°C. Deletion of *BRG1*, *ROB1*, *TEC1*, *WOR1*, and *WOR3* had little effect on colony wrinkling or biofilm formation in the *efg1*Δ/Δ *ndt80*Δ/Δ double mutant background (Fig 6A). However, deletion of *BCR1*, *UME6*, and *HGC1* caused prominent reductions in colony wrinkling and biofilm formation (Fig 6A). Detailed examination of biofilm formation showed that the *bcr1*Δ/Δ, *ume6*Δ/Δ, and *hgc1*Δ/Δ mutations in the *efg1*Δ/Δ *ndt80*Δ/Δ background caused severe reductions in biofilm depth (Fig 6B side views), surface-adherent populations (Fig 6B apical views), and biofilm volumes (Fig 6C). Biofilm defects were also observed in the triple mutants at 37°C in several media (S2 Fig). Filamentation assays indicated that the *bcr1*Δ/Δ, *ume6*Δ/Δ, and *hgc1*Δ/Δ mutations did not block filamentation of the *efg1*Δ/Δ *ndt80*Δ/Δ double mutant (Fig 6D), but caused a quantitative reduction in cell unit length (Fig 6E). These results indicate that *BCR1*, *UME6*, and *HGC1* contribute to the *efg1*Δ/Δ *ndt80*Δ/Δ emergent phenotypes. Because all three genes were upregulated in the *efg1*Δ/Δ *ndt80*Δ/Δ double mutant, their expression may relay a signal generated by the *efg1*Δ/Δ *ndt80*Δ/Δ genotype that drives emergent biofilm formation.

### A shared Bcr1/Ume6 target gene in the emergent program

**Fig 7:**
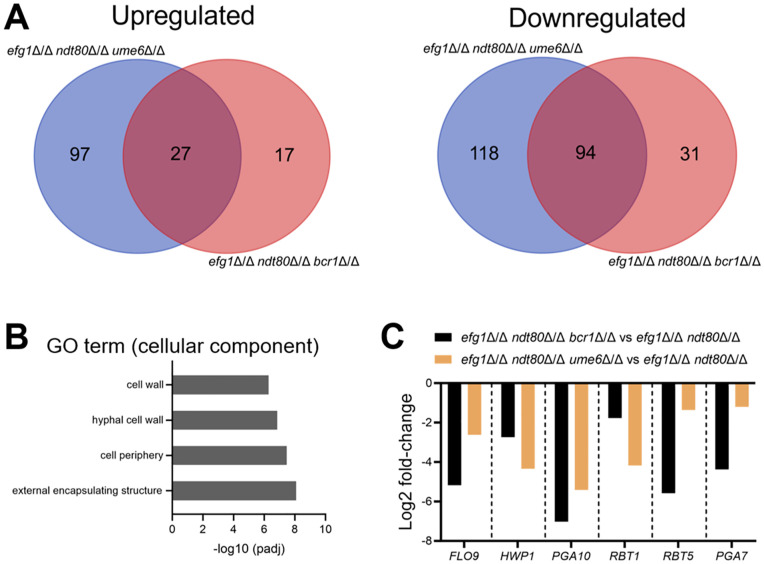
Gene expression impact of *bcr1*Δ/Δ and *ume6*Δ/Δ mutations in the *efg1*Δ/Δ *ndt80*Δ/Δ background. RNA-seq was conducted on *efg1*Δ/Δ *ndt80*Δ/Δ, *efg1*Δ/Δ *ndt80*Δ/Δ *bcr1*Δ/Δ, and *efg1*Δ/Δ *ndt80*Δ/Δ *ume6*Δ/Δ strains from the SC5314 background. Strains were grown in YPD + FBS liquid medium at 30°C for 4 hours with shaking. **(A)** Venn diagrams depicting impact of Bcr1 or Ume6 in the *efg1*Δ/Δ *ndt80*Δ/Δ mutant. Numbers in the Venn diagram indicates genes that were significantly differentially expressed between the strains indicated (log2 fold-change of greater than 1 or less than -1, padj < 0.05). **(B)** The bar graph depicts p-values from Gene Ontology term analysis of sets of genes that had significantly lower expression in both *efg1*Δ/Δ *ndt80*Δ/Δ *bcr1*Δ/Δ and *efg1*Δ/Δ *ndt80*Δ/Δ *ume6*Δ/Δ compared to the wild-type strain. **(C)** A bar graph depicts select cell surface protein gene expression levels in *efg1*Δ/Δ *ndt80*Δ/Δ *bcr1*Δ/Δ and *efg1*Δ/Δ *ndt80*Δ/Δ *ume6*Δ/Δ compared to the *efg1*Δ/Δ *ndt80*Δ/Δ mutant (log2 fold-change of greater than 1 or less than -1, padj < 0.05).

We hypothesized that Bcr1 and Ume6 may share downstream target genes that contribute to emergent biofilm formation. To find candidate targets we conducted RNA-seq on *efg1*Δ/Δ *ndt80*Δ/Δ, *efg1*Δ/Δ *ndt80*Δ/Δ *bcr1*Δ/Δ, and *efg1*Δ/Δ *ndt80*Δ/Δ *ume6*Δ/Δ strains grown in YPD + FBS at 30°C for 4 hours. The data indicated that the *efg1*Δ/Δ *ndt80*Δ/Δ *bcr1*Δ/Δ and *efg1*Δ/Δ *ndt80*Δ/Δ *ume6*Δ/Δ triple mutants had 94 shared downregulated genes and 27 shared upregulated genes relative to the double mutant (Fig 7A, S1 Table). Gene ontology analysis of the common downregulated genes showed enrichment for cell surface-associated categories, including external encapsulating structure and cell periphery (Fig 7B). Downregulation of a sampling of cell surface protein genes is shown in Fig 7C. Notably, this group included the putative adhesin gene *FLO9*, a member of the *HYR/IFF* family [28], which also had been upregulated in the *efg1*Δ/Δ *ndt80*Δ/Δ mutant relative to the wild type (Fig 5A). We considered the hypothesis that diminished expression of surface protein genes contributes to the observed biofilm defects upon loss of Bcr1 or Ume6.

**Fig 8:**
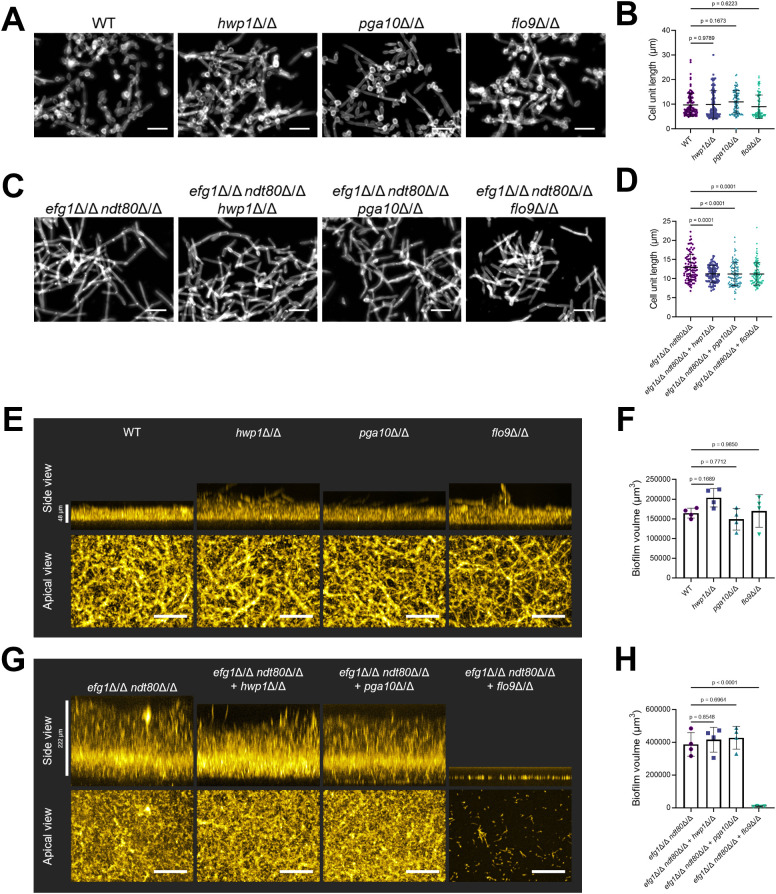
*FLO9* function in emergent biofilm formation. **(A and C)** For filamentation assays, cells were grown in YPD + FBS at 30°C for 4 hours and stained with Calcofluor-White. The white scale bar indicates 20 microns. **(B and D)** The scatter plot indicates cell unit length of each strain. Cell lengths were measured using a minimum of 100 cells from 3 different fields. The images represent three independent experiments. Statistical significance was determined using one-way ANOVA (Tukey’s multiple comparisons test). The line and error bars indicate the mean ±  s.d. Scale bars in the apical-view images indicate 100 microns. **(E and G)** For biofilm formation, cells were grown in YPD + FBS at 30°C for 24 hours and stained with Calcofluor-White. Side-view projection and apical view images are shown. Scale bars in the apical-view images indicate 100 microns. **(F and H)** Biofilm volume measurements. Statistical significance was determined using one-way ANOVA (Tukey’s multiple comparisons test). Values are the mean ±  s.d of four biological replicates.

To test functions of shared Bcr1-Ume6 target genes, we focused on three genes that specify cell surface-associated products: *HWP1*, *PGA10*, and *FLO9*. *HWP1* and *PGA10* are required for conventional biofilm formation [29,30]; *FLO9* is largely uncharacterized but predicted to encode an adhesin. We deleted each gene in the wild-type and *efg1*Δ/Δ *ndt80*Δ/Δ double mutant backgrounds. Deletion of these genes in the wild-type background did not affect filamentation under our conditions (Fig 8A, 8B). Deletion of these genes in the *efg1*Δ/Δ *ndt80*Δ/Δ background caused a mild though statistically significant reduction in filamentous cell length (Fig 8C, 8D). These results indicate that *HWP1*, *PGA10*, and *FLO9* are not major determinants of filamentation in this context.

We then examined biofilm formation in YPD + FBS at 30°C. Deletions of *HWP1* and *PGA10* did not cause a biofilm defect in the wild-type (Fig 8E, 8F) or *efg1*Δ/Δ *ndt80*Δ/Δ double mutant backgrounds (Fig 8G, 8H) under these conditions. Deletion of *FLO9* also caused no biofilm defect in the wild-type background (Fig 8E, 8F). However, deletion of *FLO9* caused a severe biofilm defect in the *efg1*Δ/Δ *ndt80*Δ/Δ double mutant background (Fig 8G, 8H), as observed with two independent *efg1*Δ/Δ *ndt80*Δ/Δ *flo9*Δ/Δ isolates (S3 Fig). These results indicate that *FLO9* is required for emergent biofilm formation.

## Discussion

Biofilm formation is critical for *C. albicans* commensalism and pathogenicity. This central role is reflected in the large number of regulatory and effector genes that govern biofilm-related phenotypes. A small group of transcription factors, often called the master regulators [2,31], has been spotlighted because they are required for biofilm formation under numerous conditions. If deletion of one master regulator gene causes a severe biofilm defect, we would expect the deletion of two master regulator genes to cause a severe biofilm defect as well. The study we presented here was prompted when that expectation was not met.

**Fig 9:**
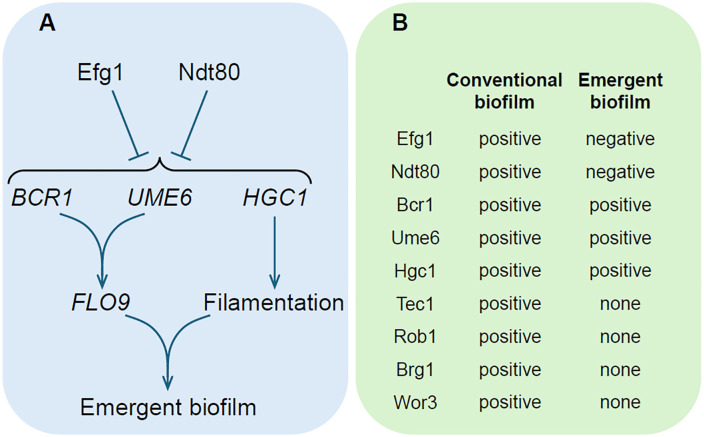
Emergent biofilm regulation. **(A)** The transcription factors Efg1 and Ndt80 each repress emergent biofilm formation. Efg1 and Ndt80 act by repressing transcription factor genes *BCR1* and *UME6* and hyphal cyclin gene *HGC1*. Bcr1 and Ume6 are both required to activate putative adhesin gene *FLO9*; Hgc1 is required for filamentation. **(B)** The table summarizes roles of select regulators in conventional biofilm formation, which is observed in otherwise wild-type strains, and in emergent biofilm formation, which is observed in *efg1*Δ/Δ *ndt80*Δ/Δ double mutants.

We defined here a process of biofilm formation and filamentation that occurs in *efg1*Δ/Δ *ndt80*Δ/Δ double mutants (Fig 9A). We refer to it as an emergent biofilm/filamentation program because the double mutant phenotypes could not have been predicted from the single mutant phenotypes. It has some features that are shared with the well-studied conventional biofilm/filamentation program (Fig 9B). For example, both biofilm programs depend upon Hgc1, Bcr1, and Ume6 under many growth conditions. The emergent biofilm/filamentation program also has features that distinguish it from the conventional biofilm/filamentation program (Fig 9B). Perhaps the most striking difference is that the emergent program occurs in the absence of most master regulators, including Efg1, Ndt80, Brg1, Rob1, and Tec1. Also, emergent biofilms are dependent upon prospective adhesin gene *FLO9*, and conventional biofilms are not. These comparisons indicate that the emergent biofilm/filamentation program is a variation on the theme of the conventional program, rather than an entirely independent process.

From one standpoint, Efg1 and Ndt80 seem functionally similar: each is required for filamentation and biofilm formation and for full expression of several biofilm/hypha-associated genes [8,15,21–23]. They are both among the biofilm master regulators that bind to one another’s gene regulatory regions and govern one another’s expression [8]. The existence of synthetic *efg1*Δ/Δ *ndt80*Δ/Δ phenotypes is consistent with the idea that Efg1 and Ndt80 have a shared function, though the emergent phenotypes themselves are unexpected. However, we note that *efg1*Δ/Δ and *ndt80*Δ/Δ mutations each cause distinct phenotypes, an indication that Efg1 and Ndt80 each have some unique functions. For example, in the extensive parallel comparisons of Homann et al. [32], the *efg1*Δ/Δ mutant presented better growth than the wild type on plates containing 5-fluorocytosine or copper, while the *ndt80*Δ/Δ mutant presented weaker growth; the *efg1*Δ/Δ mutant presented normal sensitivity to fluconazole, while the *ndt80*Δ/Δ mutant was hypersensitive; the *efg1*Δ/Δ mutant had reduced agar invasion, while the *ndt80*Δ/Δ mutant had increased agar invasion. In terms of gene expression impact, only ~10% of genes with altered expression in an *efg1*Δ/Δ mutant have altered expression in an *ndt80*Δ/Δ mutant [8]. And while both Efg1 and Ndt80 are found in complexes with Ume6, the effects on Ume6-DNA binding of *efg1*Δ/Δ and *ndt80*Δ/Δ mutations are distinguishable [24]. These measures argue that there is only partial overlap among Efg1 and Ndt80 functions.

The emergent phenotypes vary in severity among *C. albicans* clinical isolates. Strain differences in the phenotypic impact of a mutation are well known in *C. albicans* [33,34] and in every organism [25]. For *efg1*Δ/Δ *ndt80*Δ/Δ mutants, the SC5314 background presented more prominent emergent phenotypes than the other backgrounds tested. Why might that be? It is known that SC5314 has an unusual *ROB1* allele that drives filamentation and biofilm formation [35]; that allele is not present in the other strains we examined [18]. However, that *ROB1* allele cannot be responsible for the unique ability of SC5314 *efg1*Δ/Δ *ndt80*Δ/Δ mutants to produce biofilm, because a *rob1*Δ/Δ mutation does not block biofilm formation in the SC5314 *efg1*Δ/Δ *ndt80*Δ/Δ strain. For the four strains P76067, P57055, P87, and P75010, we suggest that emergent phenotypes are less prominent than in SC5314 because those strains have lower RNA levels of *HGC1* than SC5314 [17]. In addition, the three strains P76067, P57055, and P75010 have lower RNA levels of *BCR1, FLO9,* and *UME6* than SC5314 [17]. Therefore, a simple hypothesis is that SC5314 presents stronger *efg1*Δ/Δ *ndt80*Δ/Δ emergent phenotypes than other strains because it has higher expression levels of one or more of the genes required for the emergent phenotypes.

Mutant analysis shows that *FLO9* is required for emergent biofilm formation. *FLO9* belongs to the *HYR/IFF* adhesin gene family [36,37], which is found in all *Candida* pathogens [38,39]. Flo9 is required for adherence to the oral pathogen *Fusobacterium nucleatum* [40]. A recent study shows that the *C. albicans*-*F. nucleatum* pair is found among colorectal cancer patient microbiota, where this colonization is predictive of poor outcomes [41]. Interestingly, the worst outcomes are associated with strong hyphal growth and elevated *FLO9* expression in the *C. albicans* isolates [41]. An interesting though speculative possibility is that the emergent biofilm/filamentation program is activated in those colorectal cancer *C. albicans* isolates.

Why does *C. albicans* have this emergent biofilm/filamentation program? One idea is related to the evolutionary origin of *C. albicans* biofilm formation. The circuitry that governs conventional biofilm formation is complex, with six master regulators that can control one another’s expression [2,31], plus growing evidence for protein-protein interactions among master regulators [14]. We speculate that the emergent biofilm program, with what seems to be a stripped-down regulatory network, may have been the starting point to which additional regulators and effectors were added.

A second idea is that the emergent program serves as a backup system that may be deployed under particular environmental conditions. Processes that are central to *C. albicans* survival are expected to have built-in redundancy [42,43]. The ability of *C. albicans* to inhabit a broad range of body sites [44,45] may reflect a balance of activities of multiple biofilm programs.

Perhaps the most striking feature of the emergent biofilm program is that it is switched on by the loss of function of two biofilm master regulators, Efg1 and Ndt80. The role of Efg1 we describe, as a co-negative regulator of biofilm formation and filamentation, is especially surprising because Efg1 is required for both processes under almost all conditions examined in decades of prior studies [15]. The one exception of which we are aware is that an *efg1*Δ/Δ mutant presents increased filamentation compared to the wild type under hypoxic conditions [12], such as when cells are embedded in agar [16]. Hypoxic filamentation and emergent biofilm formation are similar in that they are repressed by Efg1, but they have several distinguishing features. Hypoxic filamentation depends upon Brg1 [12]; emergent biofilm formation does not. Hypoxic filamentation is repressed by Bcr1 [12]; emergent biofilm formation is not. (Rather, emergent biofilm formation depends upon Bcr1). Hypoxic filamentation of an *efg1*Δ/Δ mutant does not occur at 37°C [12]; emergent biofilm formation does occur at 37°C. Finally, we note that Efg1 is required for hypoxic biofilm formation [46], though not hypoxic filamentation [12]; Efg1 is not required for emergent biofilm formation. Therefore, hypoxic filamentation and emergent biofilm formation are related processes in that they are repressed by Efg1, but there are numerous differences between the processes.

Repression and activation of the same targets by Efg1 can occur in different environments, as described above, and also in different genetic backgrounds [11,19]. A comparison among five *C. albicans* strains showed that many direct Efg1 targets may be activated by Efg1 in one strain and repressed by Efg1 in another [11]. Well-known genes with such strain-dependent regulation include *ECM331* and *HYR1*. Increased dosage of transcription factor genes *BRG1*, *TEC1*, or *WOR1* were found to change effects of an *efg1*Δ/Δ mutation – upregulation versus downregulation – on several direct Efg1 target genes [11]. These prior studies argue that Efg1 is poised to repress or activate some genes, depending on the balance of functionally related transcription factors.

Molecular studies of Efg1 indicate that it functions in phase-separated condensates that can include other biofilm regulators [13,14]. Ndt80 has a prion-like domain that could allow it to participate in condensate formation, though this property has not been established empirically [14]. Perhaps the composition of Efg1- or Ndt80-containing complexes may affect their activity to yield activation or repression. One simple idea is that Efg1 may activate biofilm-relevant target genes in the presence of Ndt80, but repress them in the absence of Ndt80. This explanation, while speculative, has a precedent in mammalian Dual-Role Transcription Factors [47]. These are condensate-forming developmental regulators that can function as either repressors or activators.

It is surprising that a process that occurs in *efg1*Δ/Δ *ndt80*Δ/Δ double mutants would depend upon Ume6. We found recently that Ume6 forms protein complexes with Efg1 and Ndt80, and that Ume6 binding to many target promoters is improved dramatically by presence of Efg1 or Ndt80 [24]. How can Ume6 function in an *efg1*Δ/Δ *ndt80*Δ/Δ double mutant then? Ume6 also functions in protein complexes with Upc2 [24], so in theory a Ume6-Upc2 complex could drive emergent biofilm formation and filamentation. We also note that many Ume6 genomic binding targets lack evident Efg1, Ndt80, or Upc2 recognition motifs [24]. It is possible that Ume6 has additional binding partners yet to be discovered.

## Materials and methods

### Ethics statement

All animal experiments were conducted in compliance with the guidelines and regulations set forth by the Animal Care and Use Committee of the University of Wisconsin. The present study was approved by the Animal Care and Use Committee of the University of Wisconsin.

### Strains and media

Strains used in this study were maintained in 15% glycerol frozen stocks at -80°C. Prior to use, cells were routinely grown on YPD agar plates (2% dextrose, 2% Bacto peptone, 1% yeast extract, 2% Bacto agar) for overnight at 30°C and then cultured in liquid YPD medium overnight at 30°C with agitation. Transformants were selected on YPD plus 400 μg/mL nourseothricin (clonNAT; Gold Biotechnology) or complete synthetic medium (2% dextrose, 1.7% Difco yeast nitrogen base with ammonium sulfate and auxotrophic supplements). All strains used in this study are listed in S2 Table in the supplemental material, including those from previous studies [19,23].

### Strain constructions

To manipulate *C. albicans* genome, the transient clustered regularly interspaced short palindromic repeat (CRISPR) and CRISPR-associated gene 9 (CRISPR-Cas9) system was employed as previously described in detail [48]. Generally, the Cas9 cassette was amplified from the plasmid pV1093, and each of single guide RNA (sgRNA) cassette was generated by using split-joint PCR with “sgRNA/F YFG1” and “SNR52/R YFG1” as previously described in detail [19]. We use “YFG1” here as the acronym for Your Favorite Gene 1; in the actual primers the relevant gene name is used in place of “YFG1.” Primers and plasmids used, including those from previous studies [24,48–52], are listed in S3 Table.

To construct the *efg1*Δ/Δ *ndt80*Δ/Δ double mutant, the *NDT80* deletion cassette was amplified from the plasmid pNAT with primers ‘Ndt80/F_NAT’ and ‘Ndt80/R_NAT’ and integrated in the *efg1*Δ/Δ mutant which is a NAT senstive strain. The *NDT80* sgRNA DNA cassette was generated using split-joint PCR with primers ‘sgRNA/F_NDT80’ and ‘SNR52/R_NDT80’. Transformants were screened on YPD containing 400 μg/mL Nourseothricin (NAT), and candidates were genotyped by PCR using primers ‘Ndt80 check up/F’ and ‘Ndt80 check int/R) for absence of *NDT80* ORF and using primers ‘Ndt80 check up/F’ and ‘NAT_CRIME_R2’ for the presence of the NAT marker at the *NDT80* locus.

A histidine auxtroph *efg1*Δ/Δ *ndt80*Δ/Δ double mutant was generated in the *ndt80*Δ/Δ mutant which is a NAT sensitive strain. The *EFG1* deletion cassette was amplified from the plasmid pNAT with primers ‘EFG1-NAT_F’ and ‘EFG1-NAT_R’. The *NDT80* sgRNA DNA cassette was generated using split-joint PCR with primers ‘sgRNA/F_EFG1-2’ and ‘SNR52/R_EFG1-2’, and the r1 sgRNA DNA cassette was generated with primers ‘sgRNA/F r1’ and ‘sgRNA/R r1’. Transformants were screened on YPD containing 400 μg/mL Nourseothricin (NAT) and replica plated onto CSM media lacking histidine to screen for histidine axutrophy. Candidates were genotyped by PCR using primers ‘Efg1 check up/F’ and ‘Efg1 check int/R) for absence of *EFG1* ORF and using primers ‘Efg1 check up/F’ and ‘NAT_CRIME_R2’ for the presence of the NAT marker at the *EFG1* locus.

To delete genes, including *BCR1*, *BRG1*, *HGC1*, *RME1*, *ROB1*, *TEC1*, *UME6*, *WOR1*, *WOR3*, *HWP1*, *PGA10*, and *FLO9*, in the *efg1*Δ/Δ *ndt80*Δ/Δ double mutant background, each deletion cassette for *BCR1*, *BRG1*, *HGC1*, *RME1*, *TEC1*, *UME6*, *WOR1* and *WOR3* was amplified as previously decribed [11,17,19,53,54]. The deletion cassette for *ROB1* was amplified using genomic DNA from the *rob1*Δ/Δ mutant. The primer pairs used were ‘Rob1 check/F’ and ‘HIS_CRIME/R’ or ‘Rob1 check/R’ and ‘HIS_CRIME/F’ [32]. Transformants were screened on CSM media lacking histidine. Candidates were genotyped by PCR using primers ‘check up/F’ and ‘check int/R’ for absence of each gene ORF and using primers ‘check up/F’ and ‘HIS_CRIME_R’ for the presence of the *CdHIS1* marker at each gene locus.

To delete genes including *HWP1*, *PGA10*, and *FLO9* in both the wild type and the *efg1*Δ/Δ *ndt80*Δ/Δ double mutant, the deletion cassette was amplified from the plasmid pMH1 with primers ‘HIS1 CRIME/F’ and ‘YFG1 del rHIS1r-KpnI/R’ or the plasmid pMH2 with primers ‘YFG1 del rHIS1r-SapI/F’ and ‘HIS1 CRIME/R’. The sgRNA DNA cassette for each gene was generated using split-joint PCR with primers ‘sgRNA/F_YFG1’ and ‘SNR52/R_YFG1’. (Again, “YFG1” is a placeholder for the actual gene name that appears in S3 Table.) Transformants were screened on CSM media lacking histidine. Candidates were genotyped by PCR using primers ‘check up/F’ and ‘check int/R’ for absence of each gene ORF and using primers ‘check up/F’ and ‘HIS_CRIME_R’ for the presence of the *CdHIS1* marker at each gene locus.

To construct *NDT80* complemented strain in the *efg1*Δ/Δ *ndt80*Δ/Δ double mutant, the *NDT80-FLAG-CdHIS1* cassette was amplifed using genomic DNA from the Ndt80-FLAG strain [24]. The *NDT80* ORF region was generated with primers ‘Ndt80 check/F’ and ‘Act1 term int/R’, and the *FLAG-CdHIS1* region was generated with primers ‘Ndt80_orf_int/F’ and ‘Ndt80_check/R’. The r1 sgRNA DNA cassette was generated with primers ‘sgRNA/F r1’ and ‘sgRNA/R r1’. These cassettes were then used to integrate the complementation cassette into the original locus of *NDT80*. Transformants were screened on CSM media lacking histidine. Candidates were genotyped by PCR using primers ‘Ndt80_far_check up/F’ and ‘Ndt80 int/R’ for presence of *NDT80* ORF and using primers ‘Ndt80 orf far check/F’ and ‘HIS_CRIME_R’ for the presence of the *CdHIS1* marker at *NDT80* locus.

### Filamentation assay

To assay hyphal formation in *C. albicans* strains, cell culture and fixation were performed according to previous published methods [19]. Cells were grown in YPD overnight at 30°C and transferred to indicated pre-warmed medium at a cell density adjusted to an optical density at 600 nm (OD600) of 0.5. Cells were incubated in a glass test tube for 4 hours at 30°C and fixed with 4% formaldehyde in phosphate-buffered saline (PBS) for 15 min. Calcofluor-white was used for cell staining, and cells were observed using a Zeiss Axio observer Z.1 fluorescence microscope with a 20X 0.8- numerical-aperture (NA).

### Biofilm assays

Biofilm production and imaging were followed previously published methods with minor modifications [11,19]. Briefly, cells were grown in YPD overnight at 30°C and the harvested cells were washed with MilliQ-filtered sterile water. Then cells were transferred to indicated pre-warmed medium to achieve an OD600 of 0.05 in 96-well plate (Greiner, 655090) and incubated for 24 hours at 30°C. Biofilms were fixed with 4% formaldehyde, washed with PBS twice and stained with Calcofluor-white (200 μg/mL in PBS), then 2,2′-Thiodiethanol (TDE) in PBS was added to each well for clarification and refractive index matching. Each biofilm sample was prepared as a biological triplicate and observed using a Keyence fluorescence microscope (BZ-X800E) with a Keyence 20X objective and 2X zoom.

In vivo biofilm assays in the rat venous catheter model were conducted as described by Andes et al. [26].

### RNA extraction and sequencing

Cells were grown in YPD at 30°C for overnight and washed with MilliQ-filtered sterile water once. For RNA extraction from planktonic conditions, cells were inoculated into 25 mL of YPD medium + 10% fetal bovine serum (YPD + 10% FBS) to an OD600 of 0.2 and incubated at 30°C for 4 hours with shaking at 225 rpm. Then cells were harvested using vacuum filtration and quickly frozen at -80°C. RNA extraction, library preparation, and RNA sequencing were performed according to previously described methods [19].

### qRT-PCR

RNA samples were prepared from three biological replicates of each strain, and 1 μg of total RNA per sample was used for cDNA synthesis using iScript gDNA clear cDNA synthesis kit (Bio-Rad, 1725035) with diluted cDNA, following manufacturer’s instruction. Then, qRT-PCR was conducted using iQ SYBR Green Supermix (Bio-Rad, 1708880). The *CDC28* RNA was used as a control to normalize tested genes in each experiment using the threshold cycle ΔΔCT method.

### Data Interpretation

Interpretations and hypotheses were guided by the comprehensive information at the *Candida* Genome Database [7].

### Data analysis software

Biofilm and filamentation images were processed using Image J (Fiji) [55]. Statistical analyses and graph generations were carried out using GraphPad Prism version 9 (GraphPad Software, Inc., La Jolla). Volcano plots were generated using VolcaNoseR (https://huygens.science.uva.nl/VolcaNoseR2/).

## Supporting information

S1 Fig The hyper-filamentous phenotype in the *efg1*Δ/Δ *ndt80*Δ/Δ mutant is suppressed by complementation of the *NDT80* allele. (A) For filamentation assay, cells were grown in YPD + FBS at 30°C or 37°C for 4 hours and stained with Calcofluor-White. The white scale bar indicates 20 microns. (B) For biofilm formation, cells were grown in YPD + FBS at 30°C for 24 hours and stained with Calcofluor-White. Side-view projection and apical view images are shown.
(PDF)

S2 Fig Deletion of *BCR1*, *UME6*, or *HGC1* in the *efg1*Δ/Δ *ndt80*Δ/Δ mutant results in defective emergent biofilm formation at 37°C. For biofilm formation, cells were grown in YPD, YPD + FBS, Spider, RPMI, or RPMI + FBS at 37°C for 24 hours and stained with Calcofluor-White.
(PDF)

S3 Fig Biofilm formation in two independent *efg1*Δ/Δ *ndt80*Δ/Δ *flo9*Δ/Δ isolates. For biofilm formation, cells were grown in YPD + FBS at 30°C for 24 hours and stained with Calcofluor-White.
(PDF)

S1 Table RNA-seq data. This table provides Log2FC and padj from RNA-seq analysis, and in a second tab, a list of differentially expressed genes for each comparison.
(XLSX)

S2 Table Strains used in this study. This table provides information about *C. albicans* strains used, including the strain name, the genotype, some notes about special features, and references for strains used in previous publications.
(XLSX)

S3 Table Primers and plasmids used in this study. This table provides information about oligonucleotide primers (primer name, sequence) and, in a separate tab, plasmids (plasmid name, description, bacterial marker, and references) used in this study.
(XLSX)
