# Supplementary material for: An emergent biofilm program from inactivation of Candida albicans master regulators Efg1 and Ndt80
Source: PLoS Pathog. 2026 Jul 20;22(7):e1014469. doi: 10.1371/journal.ppat.1014469 (PMC13399525; doi:10.1371/journal.ppat.1014469)
Supplement: S1 Fig — (A) For filamentation assay, cells were grown in YPD + FBS at 30°C or 37°C for 4 hours and stained with Calcofluor-White. The white scale bar indicates 20 microns. (B) For biofilm formation, cells were grown in YPD + FBS at 30°C for 24 hours and stained with Calcofluor-White. Side-view projection and apical view images are shown. (PDF) [file ppat.1014469.s001.pdf]

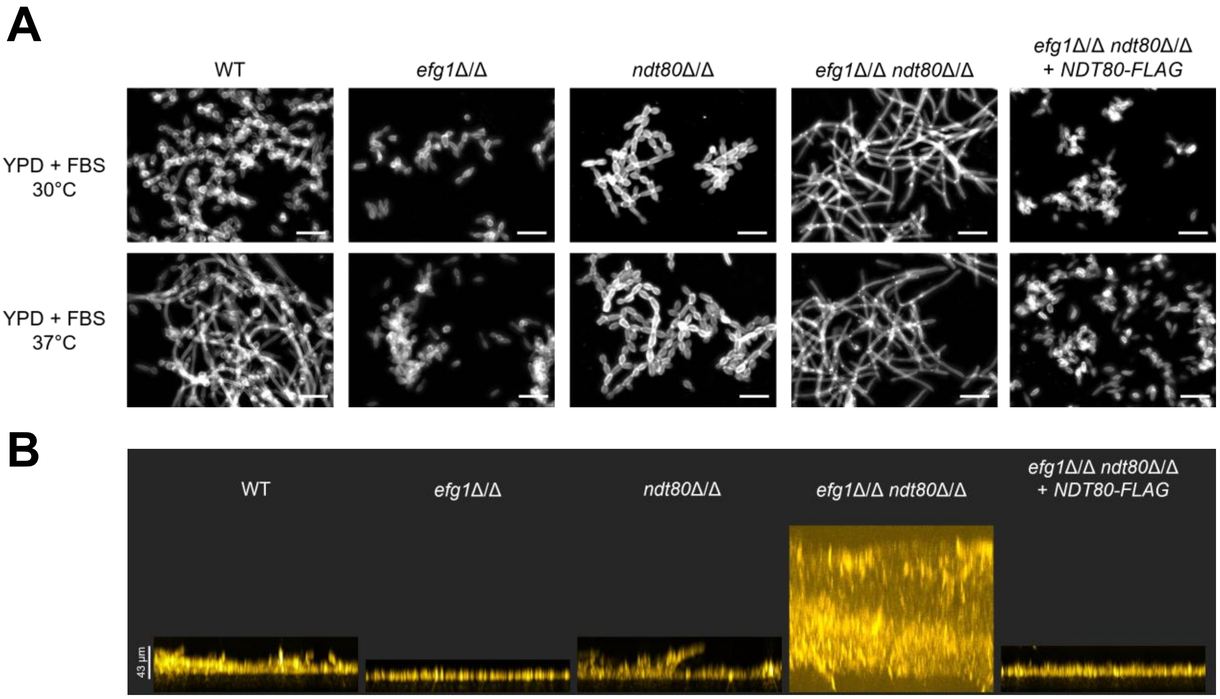

Figure S1. The hyper-filamentous phenotype in the *efg1*Δ/Δ *ndt80*Δ/Δ mutant is suppressed by complementation of the *NDT80* allele.
(A) For filamentation assay, cells were grown in YPD + FBS at 30°C or 37°C for 4 hours and stained with Calcofluor-White. White scale bar indicates 20 microns.
(B) For biofilm formation, cells were grown in YPD + FBS at 30°C for 24 hours and stained with Calcofluor-White. Side-view projection and apical view images are shown.
