# Supplementary material for: An emergent biofilm program from inactivation of Candida albicans master regulators Efg1 and Ndt80
Source: PLoS Pathog. 2026 Jul 20;22(7):e1014469. doi: 10.1371/journal.ppat.1014469 (PMC13399525; doi:10.1371/journal.ppat.1014469)
Supplement: S2 Fig — For biofilm formation, cells were grown in YPD, YPD + FBS, Spider, RPMI, or RPMI + FBS at 37°C for 24 hours and stained with Calcofluor-White. (PDF) [file ppat.1014469.s002.pdf]

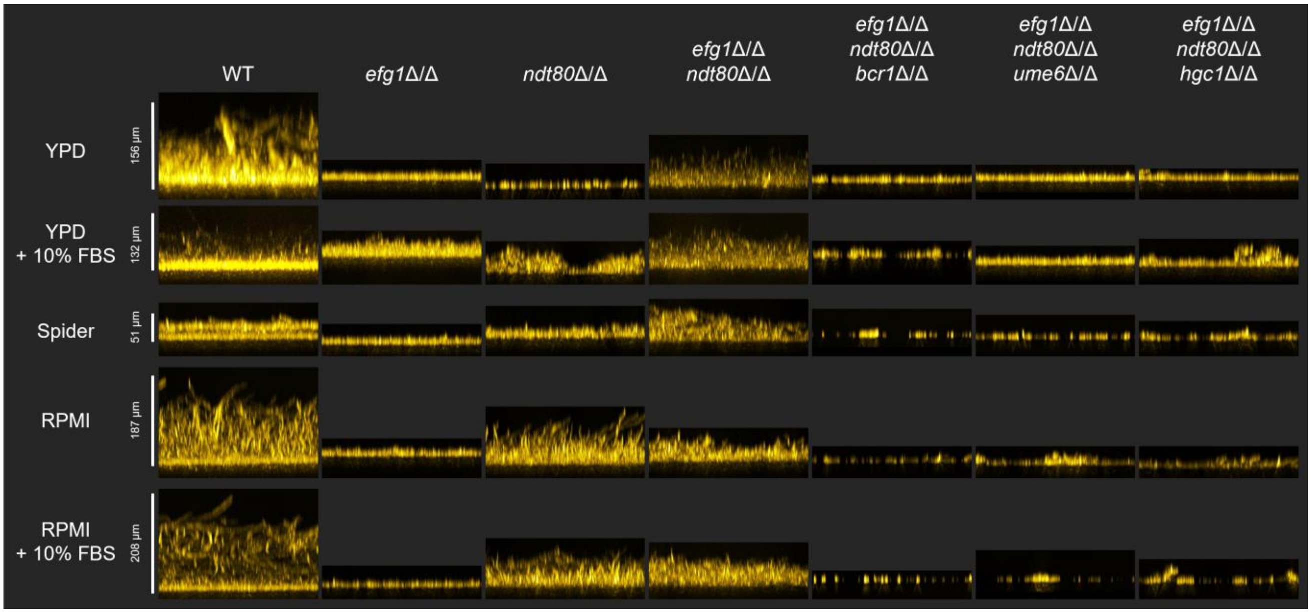


**Figure S2. Deletion of *BCR1*, *UME6*, or *HGC1* in the *efg1Δ/Δ ndt80Δ/Δ* mutant results in defective emergent biofilm formation at** 37°C **.**
For biofilm formation, cells were grown in YPD, YPD + FBS, Spider, RPMI, or RPMI + FBS at 37°C for 24 hours and stained with Calcofluor-White.
