# Supplementary material for: An emergent biofilm program from inactivation of Candida albicans master regulators Efg1 and Ndt80
Source: PLoS Pathog. 2026 Jul 20;22(7):e1014469. doi: 10.1371/journal.ppat.1014469 (PMC13399525; doi:10.1371/journal.ppat.1014469)
Supplement: S3 Fig — For biofilm formation, cells were grown in YPD + FBS at 30°C for 24 hours and stained with Calcofluor-White. (PDF) [file ppat.1014469.s003.pdf]

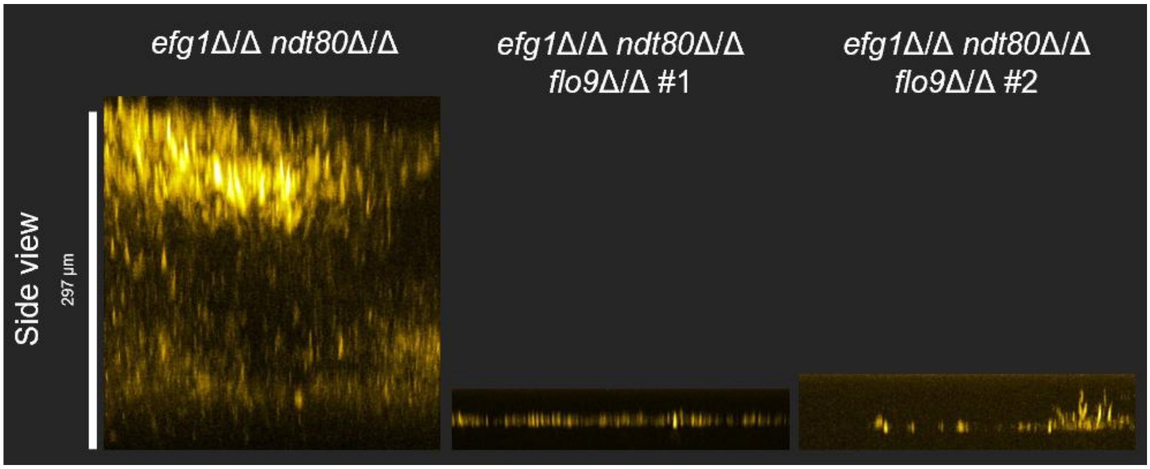


**Figure S3. Biofilm formation in two independent *efg1*Δ/Δ *ndt80*Δ/Δ *flo9*Δ/Δ isolates.**
For biofilm formation, cells were grown in YPD + FBS at 30°C for 24 hours and stained with Calcofluor-White.
