# Supplementary material for: An emergent biofilm program from inactivation of Candida albicans master regulators Efg1 and Ndt80
Source: PLoS Pathog. 2026 Jul 20;22(7):e1014469. doi: 10.1371/journal.ppat.1014469 (PMC13399525; doi:10.1371/journal.ppat.1014469)
Supplement: Attachment — Submitted filename: Response to Reviewers.docx [file ppat.1014469.s008.docx]

**Reviewers' Comments:**

Reviewer's Responses to Questions

**Part I - Summary**
Please use this section to discuss strengths/weaknesses of study, novelty/significance, general execution and scholarship.

Reviewer #1: The submission from Do et al considers the complex genetic determinants of biofilm formation in the medically important fungal pathogen Candida albicans. Filamentation and biofilm formation are important phenotypes of many fungal pathogens that enable them to avoid certain aspects of the host immune response and to resist clearance by antibiotics. These properties can represent significant problems with catheterization or implant surgeries. Therefore, understanding the molecular genetic underpinnings of this process could provide significant insights into the causes and prevention of serious fungal infections. Previous work had identified two key regulators of the gene expression cascade the leads to filamentation and biofilm formation, Efg1 and Ndt80. Disruption of the genes encoding these transcription factors prevents the formation of biofilms. Remarkably, in this manuscript the authors surprisingly report that creation of the double knockout rescues both filamentation and biofilm formation, and even enhances these phenotypes under certain conditions, including biofilm formation in a mouse catheterization model. The authors go on to characterize many aspects of the knockout lines, including gene expression profiles and interactions with other loci previously shown to be important for these phenotypes. The properties described here are somewhat strain specific and the paper does not solve what is clearly an exceptionally complex regulatory hierarchy, nonetheless the manuscript provides new insights into the gene expression cascades that determine biofilm formation and adds to our understanding of the regulatory network of transcription factors controlling it.

Thank you for this concise and supportive summary!

Reviewer #2: This manuscript reports on new mechanistic insight into C. albicans biofilm formation, which is significant because biofilms commonly underlie the development of lethal systemic infections. The key result of this manuscript is the surprising discovery that combining two mutations (efg1 and ndt80) that individually cause defects in biofilm formation results in the opposite phenotype – biofilm formation that is better than either single mutant alone. This surprising genetic effect was most obvious in the SC5314 strain background, but the efg1 ndt80 combination resulted in improved hyphal growth in 5 other clinical isolates, strengthening the significance of this genetic interaction. Further genetic analysis showed that biofilm formation in the efg1 ngt1 strain was regulated by only a subset of the genes that have been identified previously to contribute to biofilm formation (BCR1, UME6, and HGC1), which correlated with the increased expression of these genes in the efg1 ndt80 strain. Interestingly, comparison of RNAseq data revealed that one of the commonly regulated genes was FLO9, a putative adhesin. Deletion of FLO9 essentially blocked biofilm in the efg1 ndt80 strain, highlighting a key role in this strain background. Altogether, these data show that a “stripped down” version of the complex biofilm circuitry is sufficient to drive biofilm formation, which now provides a new avenue for identifying regulators of this complex process.

We are very grateful for this positive appraisal!

Reviewer #3: The study by Do et al. reports the unexpected finding that the efg1Δ/Δ ndt80Δ/Δ double mutant of Candida albicans, despite the severe biofilm defects of each single mutant, is capable of forming robust biofilms in both in vitro and in vivo models. The authors define this as an “emergent biofilm” phenotype and provide evidence that it is mechanistically distinct from the conventional biofilm regulatory program. Through transcriptomic and genetic analyses, the study identifies key roles for Hgc1, Bcr1, Ume6, and the adhesin Flo9 in supporting this alternative biofilm state, while demonstrating independence from several canonical biofilm regulators, including Brg1, Rob1, Tec1, and Wor3. Overall, the work reveals an unexpected plasticity in the C. albicans biofilm regulatory network and suggests the existence of a simplified or primordial surface colonization program. The findings are interesting and provide new insight into how fungal virulence regulatory networks can adapt and reorganize.

The point you make here about adaptation and reorganization of virulence networks is a really interesting idea! We had not really thought about the broader possibility that other networks in *C. albicans* (or in other pathogenic fungi) might present similar plasticity. It would be neat to come up with a more general strategy to assess such plasticity. Many thanks!

**Part II – Major Issues: Key Experiments Required for Acceptance**
Please use this section to detail the key new experiments or modifications of existing experiments that should be absolutely required to validate study conclusions.

Generally, there should be no more than 3 such required experiments or major modifications for a "Major Revision" recommendation. If more than 3 experiments are necessary to validate the study conclusions, then you are encouraged to recommend "Reject".

Reviewer #1: None

Whoa! Thank you!

Reviewer #2: 1. It is interesting that the authors refer to the efg1 ndt80 genetic interaction as “emergent biofilm” formation. However, for clarity the authors should also state that it is a variation on the “synthetic” genetic interactions that have been widely used to study positive and negative interactions between genes.

Excellent point! We have added this sentence on line 122 (revision numbering):

“Formation of biofilm by the double mutant represents a synthetic genetic interaction [25].”

We reference the David Botstein book from CSH press, which is a real gem.

2. The SC5314 strain differs from other clinical isolates in that it the two different alleles of ROB1 that differ in how they promote filament formation. I think it would be interesting for the authors to comment on the fact that deletion of ROB1 did not impact the efg1 ndt80 phenotype, indicating that the ROB1 alleles were not the critical difference for the SC5314 strain phenotype caused by efg1 ndt80.

Excellent point! We added a brief paragraph (line 302) to the Discussion to incorporate this comment as well as a similar issue raised by Reviewer 3. The specific point you raised is highlighted.

“The emergent phenotypes vary in severity among *C. albicans* clinical isolates. Strain differences in the phenotypic impact of a mutation are well known in *C. albicans* [33, 34] and in every organism [25]. For *efg1*Δ/Δ *ndt80*Δ/Δ mutants, the SC5314 background presented more prominent emergent phenotypes than the other backgrounds tested. Why might that be? It is known that SC5314 has an unusual *ROB1* allele that drives filamentation and biofilm formation [35]; that allele is not present in the other strains we examined [18]. However, that *ROB1* allele cannot be responsible for the unique ability of SC5314 *efg1*Δ/Δ *ndt80*Δ/Δ mutants to produce biofilm, because a *rob1*Δ/Δ mutation does not block biofilm formation in the SC5314 *efg1*Δ/Δ *ndt80*Δ/Δ strain. For the four strains P76067, P57055, P87, and P75010, we suggest that emergent phenotypes are less prominent than in SC5314 because those strains have lower RNA levels of *HGC1* than SC5314 [17]. In addition, the three strains P76067, P57055, and P75010 have lower RNA levels of *BCR1, FLO9,* and *UME6* than SC5314 [17]. Therefore, a simple hypothesis is that SC5314 presents stronger *efg1*Δ/Δ *ndt80*Δ/Δ emergent phenotypes than other strains because it has higher expression levels of one or more of the genes required for the emergent phenotypes.”

3. FLO9 was described as a “putative adhesin”. The authors should supply more information about the basis for this description. Perhaps a supplemental figure showing the structural features on which this assessment is based? Is FLO9 conserved in other biofilm-forming species?

This was a great suggestion. We did not add a figure since the *HYR/IFF* family to which *FLO9* belongs has been well characterized. However, we added this paragraph to the Discussion (line 320) which perhaps jazzes things up:

“Mutant analysis shows that *FLO9* is required for emergent biofilm formation. *FLO9* belongs to the *HYR/IFF* adhesin gene family [36, 37], which is found in all *Candida* pathogens [38, 39]. Flo9 is required for adherence to the oral pathogen *Fusobacterium nucleatum* [40]. A recent study shows that the *C. albicans*-*F. nucleatum* pair is found among colorectal cancer patient microbiota, where this colonization is predictive of poor outcomes [41]. Interestingly, the worst outcomes are associated with strong hyphal growth and elevated *FLO9* expression in the *C. albicans* isolates [41]. An interesting though speculative possibility is that the emergent biofilm/filamentation program is activated in those colorectal cancer *C. albicans* isolates.”

4. The NCBI SRA accession number PRJNA1453028 did not work. Please check it is correct.

It works now – thanks for pointing this out!

Reviewer #3: Major comments:
1. Is Flo9 overexpression sufficient to drive biofilm formation in the efg1 or ndt80 single mutants?

This is a great suggestion! However, we have not made the relevant strains. My lab just moved into new space, and it will take some time before we are up and running. I hope that it is acceptable for us to omit this experiment.

2. Does the deletion of the Ume6 interacting protein, Upc2, affect the emergent biofilm formation? That would tell whether new binding partners are required.

Excellent point! Yes, we have done this, but it raises some complications as I explain below, and we hope to save it for another paper. First the data. Here is a complete panel of triple mutant strains assayed as in Figure 6A. The *efg1*Δ/Δ *ndt80*Δ/Δ *upc2*Δ/Δ strain is at the right side:

You can see that the *upc2*Δ/Δ mutation causes an intermediate phenotype; it blocks biofilm formation but not wrinkling. Given that Upc2 is required for hypoxic growth, and our biofilm assay definitely requires hypoxic functions, it is not entirely clear whether Upc2 is required for the larger emergent program or simply for hypoxic adaptation. In contrast, the *bcr1*Δ/Δ and *ume6*Δ/Δ mutations block both biofilm and wrinkling, which is why we focused on them. I hope you agree that the *upc2*Δ/Δ result suggests that there are other Ume6 partners, which would drive at least the wrinkling phenotype (or that Ume6 can function without DNA binding partners). This is why we raised the issue of other potential Ume6 partners at the end of the Discussion – so in case we don’t find any, at least other labs will be encouraged to look.

We hope to leave the *upc2*Δ/Δ mutant out of this story because it is a complication, and such a digression will distract from the major points. But we do not intend to bury the result! We also have Ume6-HA ChIP-seq data in the *efg1*Δ/Δ *ndt80*Δ/Δ *upc2*Δ/Δ background. The results show clearly that Ume6 binds to many genes perfectly well in the absence of its three known partners. Our plan is to publish the ChIP-seq data together with the *efg1*Δ/Δ *ndt80*Δ/Δ *upc2*Δ/Δ phenotype, since the two results converge on the same conclusion. I hope this seems reasonable to you.

**Part III – Minor Issues: Editorial and Data Presentation Modifications**
Please use this section for editorial suggestions as well as relatively minor modifications of existing data that would enhance clarity.

Reviewer #1: Overall, the manuscript is thorough, and the data presented in a clear and easily understood manner. I have only minor comments for the authors to consider.

1. In the paragraph beginning on line 87, the authors mention that both efg1 and ndt80 knockout lines display defects in biofilm formation and filamentation, leading the reader to assume that they phenocopy each other. However, from the last sentence of this paragraph, it is clear that the two mutants share some characteristics and differ in others. For unfamiliar readers, it would be helpful to know how similar these mutant lines are. If there are additional data from previous characterizations of these single mutant lines, it would be great if the authors could provide a brief summary.

We agree that this issue is important and, as far as I can tell, has not really been emphasized anywhere else. We added this paragraph to the Discussion (line 282); see what you think.

“From one standpoint, Efg1 and Ndt80 seem functionally similar: each is required for filamentation and biofilm formation and for full expression of several biofilm/hypha-associated genes [8, 15, 21-23]. They are both among the biofilm master regulators that bind to one another’s gene regulatory regions and govern one another’s expression [8]. The existence of synthetic *efg1*Δ/Δ *ndt80*Δ/Δ phenotypes is consistent with the idea that Efg1 and Ndt80 have a shared function, though the emergent phenotypes themselves are unexpected. However, we note that *efg1*Δ/Δ and *ndt80*Δ/Δ mutations each cause distinct phenotypes, an indication that Efg1 and Ndt80 each have some unique functions. For example, in the extensive parallel comparisons of Homann et al. [32], the *efg1*Δ/Δ mutant presented better growth than the wild type on plates containing 5-fluorocytosine or copper, while the *ndt80*Δ/Δ mutant presented weaker growth; the *efg1*Δ/Δ mutant presented normal sensitivity to fluconazole, while the *ndt80*Δ/Δ mutant was hypersensitive; the *efg1*Δ/Δ mutant had reduced agar invasion, while the *ndt80*Δ/Δ mutant had increased agar invasion. In terms of gene expression impact, only ~10% of genes with altered expression in an *efg1*Δ/Δ mutant have altered expression in an *ndt80*Δ/Δ mutant [8]. And while both Efg1 and Ndt80 are found in complexes with Ume6, the effects on Ume6-DNA binding of *efg1*Δ/Δ and *ndt80*Δ/Δ mutations are distinguishable [24]. These measures argue that there is only partial overlap among Efg1 and Ndt80 functions.”

2. In the section beginning on line 163, the authors compare RNA-seq data sets from a wild type line and the efg1/ndt80 double mutant. This is, of course, both interesting and key to the analysis that the authors are presenting. However, it would also be interesting to know how the double mutant compares to each single mutant (and perhaps how the single mutants compare to each other). Ideally, the RNA-seq analyses of all these lines would be performed in parallel, however this might be prohibitive at this point. Are there preexisting RNA-seq datasets for each of the single mutant lines that could be used for comparison, even if the comparison is qualitative rather than quantitative (e.g. are similar/different pathways up/down regulated)?

This is good point. However, we hope to avoid detailed examination of other datasets because we know that Efg1-dependent gene expression changes are highly dependent on the environmental conditions (from work of Ernst, Sellam, and others). We used weird conditions for RNA-seq ourselves – YPD+FBS at 30°C – because that’s where the emergent phenotypes seemed strongest. We hope it is acceptable to capture the gene expression differences in a brief summary of data in the one paper that examines both mutants in parallel, Nobile et al., 2012. (This sentence is excerpted from the paragraph above.)

“In terms of gene expression impact, only ~10% of genes with altered expression in an *efg1*Δ/Δ mutant have altered expression in an *ndt80*Δ/Δ mutant [8].”

Reviewer #2: • Lines 63-65. Sentence unclear – missing a word?

Apologies – a word was inadvertently deleted. It is fixed now:

“Strong evidence indicates that these phase-separated condensates are necessary for activation of biofilm formation and the related process of filamentation [14].”

• Line 355 to around Line 390 – define YFG1 and YFG

Thank you for catching this. We now say on line 417:

We use “YFG1” here as the acronym for Your Favorite Gene 1; in the actual primers the relevant gene name is used in place of “YFG1.”

For the cases where we used “YFG” alone, we should have used “YFG1,” and that has now been corrected.

• Lines 411-412. Unclear sentence. What is OD0.5?

Apologies; the sentence has hopefully been clarified as follows (line 474):

“Cells were grown in YPD overnight at 30°C and transferred to indicated pre-warmed medium at a cell density adjusted to an optical density at 600 nm (OD600) of 0.5.”

• define ddH2O

Thank you for catching this. I have corrected the statement on line 484 to say:

“Briefly, cells were grown in YPD overnight at 30°C and the harvested cells were washed with MilliQ-filtered sterile water.”

Line 497 was also corrected:

“Cells were grown in YPD at 30°C for overnight and washed with MilliQ-filtered sterile water once.”

Reviewer #3: Minor comments:
1. It would be good to also include the phenotype of the single mutants in Fig. 4A, 4B, and 4C.

The single mutants for SC5314 are shown earlier Figure 3. We did not include the singles for the other strain backgrounds in these experiments, though almost all have been characterized at 37°C in our earlier papers PMID: 31323072, PMID: 36696432, PMID: 41891724. I hope this seems acceptable, given that SC5314 is the main focus of our studies in this report.

2. In Fig. 5, please add what RNA-seq conditions from other studies were used to generate 5B.

Excellent suggestion! We added these sentences to the Figure 5 legend (line 781) to remind readers of our own conditions:

“RNA-seq was conducted on the wild type and *efg1*Δ/Δ *ndt80*Δ/Δ double mutant from the SC5314 background. Strains were grown in YPD+FBS liquid medium at 30°C for 4 hours with shaking, a condition that supports emergent filamentation (see Figure 4B).”

Then we added these sentences to the end of the legend (line 791) to capture for readers the other papers’ growth conditions:

“RNA-seq analysis by Cravener et al. [17] was conducted on multiple different strains grown in RPMI+FBS medium at 37°C, and strains were compared to one another to identify gene expression features that correlated with filamentation ability. RNA-seq analysis by Azadmanesh et al. [27] was conducted on strain SC5314 in diverse filamentation-inducing liquid and solid media, including RPMI, Lee’s, Spider, or FBS, all at 37°C, and compared to cells grown in (filamentation noninducing) liquid YPD medium at 30°C.”

The suggestion to remind readers of our RNA-seq conditions also applies to Figure 7, so we added (line 820):

“RNA-seq was conducted on *efg1*Δ/Δ *ndt80*Δ/Δ, *efg1*Δ/Δ *ndt80*Δ/Δ *bcr1*Δ/Δ, and *efg1*Δ/Δ *ndt80*Δ/Δ *ume6*Δ/Δ strains from the SC5314 background. Strains were grown in YPD+FBS liquid medium at 30°C for 4 hours with shaking.”

3. It would be good to add to the discussion why the emergent biofilm of the double mutant is only in SC5314, but not in other strain background. In addition, it would be good to discuss what makes Flo9 so special to be required for emergent biofilm formation, but not other adhesins. Is the regulation of Flo9 much different compared to other adhesins?

In response to this comment and a related issue raised by Reviewer 2, we added this paragraph to the Discussion (line 302). See what you think.

“The emergent phenotypes vary in severity among *C. albicans* clinical isolates. Strain differences in the phenotypic impact of a mutation are well known in *C. albicans* [33, 34] and in every organism [25]. For *efg1*Δ/Δ *ndt80*Δ/Δ mutants, the SC5314 background presented more prominent emergent phenotypes than the other backgrounds tested. Why might that be? It is known that SC5314 has an unusual *ROB1* allele that drives filamentation and biofilm formation [35]; that allele is not present in the other strains we examined [18]. However, that *ROB1* allele cannot be responsible for the unique ability of SC5314 *efg1*Δ/Δ *ndt80*Δ/Δ mutants to produce biofilm, because a *rob1*Δ/Δ mutation does not block biofilm formation in the SC5314 *efg1*Δ/Δ *ndt80*Δ/Δ strain. For the four strains P76067, P57055, P87, and P75010, we suggest that emergent phenotypes are less prominent than in SC5314 because those strains have lower RNA levels of *HGC1* than SC5314 [17]. In addition, the three strains P76067, P57055, and P75010 have lower RNA levels of *BCR1, FLO9,* and *UME6* than SC5314 [17]. Therefore, a simple hypothesis is that SC5314 presents stronger *efg1*Δ/Δ *ndt80*Δ/Δ emergent phenotypes than other strains because it has higher expression levels of one or more of the genes required for the emergent phenotypes.”

Regarding Flo9, we added this paragraph to the Discussion (line 320), which hopefully makes it sound exciting:

“Mutant analysis shows that *FLO9* is required for emergent biofilm formation. *FLO9* belongs to the *HYR/IFF* adhesin gene family [36, 37], which is found in all *Candida* pathogens [38, 39]. Flo9 is required for adherence to the oral pathogen *Fusobacterium nucleatum* [40]. A recent study shows that the *C. albicans*-*F. nucleatum* pair is found among colorectal cancer patient microbiota, where this colonization is predictive of poor outcomes [41]. Interestingly, the worst outcomes are associated with strong hyphal growth and elevated *FLO9* expression in the *C. albicans* isolates [41]. An interesting though speculative possibility is that the emergent biofilm/filamentation program is activated in those colorectal cancer *C. albicans* isolates.”

4. Line 64: add “condensates” after “phase-separated”

Done – thank you!
