# Supplementary material for: An emergent biofilm program from inactivation of Candida albicans master regulators Efg1 and Ndt80
Source: PLoS Pathog. 2026 Jul 20;22(7):e1014469. doi: 10.1371/journal.ppat.1014469 (PMC13399525; doi:10.1371/journal.ppat.1014469)
Supplement: Peer review history

# Peer review: Decision Letter 0

**DOI:** 10.1371/journal.ppat.1014469.r001

**Authors:** Michal Olszewski, Biao Ren

20 May 2026

PPATHOGENS-D-26-01006

An emergent biofilm program from inactivation of Candida albicans master regulators Efg1 and Ndt80

PLOS Pathogens

Dear Dr. Mitchell,

Thank you for submitting your manuscript to PLOS Pathogens. After careful consideration, we feel that it has merit but does not fully meet PLOS Pathogens’s publication criteria as it currently stands. Therefore, we invite you to submit a revised version of the manuscript that addresses the points raised during the review process.

Please submit your revised manuscript by Jul 19 2026 11:59PM. If you will need more time than this to complete your revisions, please reply to this message or contact the journal office at plospathogens@plos.org. When you're ready to submit your revision, log on to https://www.editorialmanager.com/ppathogens/ and select the 'Submissions Needing Revision' folder to locate your manuscript file.

Please include the following items when submitting your revised manuscript:

* A letter that responds to each point raised by the editor and reviewer(s). You should upload this letter as a separate file labeled 'Response to Reviewers'. This file does not need to include responses to any formatting updates and technical items listed in the 'Journal Requirements' section below.

* A marked-up copy of your manuscript that highlights changes made to the original version. You should upload this as a separate file labeled 'Revised Manuscript with Track Changes'.

* An unmarked version of your revised paper without tracked changes. You should upload this as a separate file labeled 'Manuscript'.

If you would like to make changes to your financial disclosure, competing interests statement, or data availability statement, please make these updates within the submission form at the time of resubmission. Guidelines for resubmitting your figure files are available below the reviewer comments at the end of this letter.

As the corresponding author, your ORCID iD is verified in the submission system and will appear in the published article. PLOS supports the use of ORCID, and we encourage all coauthors to register for an ORCID iD and use it as well. Please encourage your coauthors to verify their ORCID iD within the submission system before final acceptance, as unverified ORCID iDs will not appear in the published article. Only the individual author can complete the verification step; PLOS staff cannot verify ORCID iDs on behalf of authors.

We look forward to receiving your revised manuscript.

Kind regards,

Biao Ren

Academic Editor

PLOS Pathogens

Michal Olszewski

Section Editor

PLOS Pathogens

Sumita Bhaduri-McIntosh

Editor-in-Chief

PLOS Pathogens

orcid.org/0000-0003-2946-9497

Michael Malim

Editor-in-Chief

PLOS Pathogens

orcid.org/0000-0002-7699-2064

**Additional Editor Comments (if provided):**

**Journal Requirements:**

If the reviewer comments include a recommendation to cite specific previously published works, please review and evaluate these publications to determine whether they are relevant and should be cited. There is no requirement to cite these works unless the editor has indicated otherwise.

1) Please provide an Author Summary. This should appear in your manuscript between the Abstract (if applicable) and the Introduction, and should be 150-200 words long. The aim should be to make your findings accessible to a wide audience that includes both scientists and non-scientists. Sample summaries can be found on our website under Submission Guidelines:

https://journals.plos.org/plospathogens/s/submission-guidelines#loc-parts-of-a-submission

2) Please upload all main figures as separate Figure files in .tif or .eps format. For more information about how to convert and format your figure files please see our guidelines:

https://journals.plos.org/plospathogens/s/figures

3) Please amend your detailed Financial Disclosure statement. This is published with the article. It must therefore be completed in full sentences and contain the exact wording you wish to be published.

1) State what role the funders took in the study. If the funders had no role in your study, please state: "The funders had no role in study design, data collection and analysis, decision to publish, or preparation of the manuscript."

2) If any authors received a salary from any of your funders, please state which authors and which funders..

If you did not receive any funding for this study, please simply state: u201cThe authors received no specific funding for this work.u201d

4) We have amended your Competing Interest statement to comply with journal style. We kindly ask that you double check the statement and let us know if anything is incorrect.

**Reviewers' Comments:**

Reviewer’s Responses to Questions

**Part I - Summary**

Please use this section to discuss strengths/weaknesses of study, novelty/significance, general execution and scholarship.

Reviewer #1: The submission from Do et al considers the complex genetic determinants of biofilm formation in the medically important fungal pathogen Candida albicans. Filamentation and biofilm formation are important phenotypes of many fungal pathogens that enable them to avoid certain aspects of the host immune response and to resist clearance by antibiotics. These properties can represent significant problems with catheterization or implant surgeries. Therefore, understanding the molecular genetic underpinnings of this process could provide significant insights into the causes and prevention of serious fungal infections. Previous work had identified two key regulators of the gene expression cascade the leads to filamentation and biofilm formation, Efg1 and Ndt80. Disruption of the genes encoding these transcription factors prevents the formation of biofilms. Remarkably, in this manuscript the authors surprisingly report that creation of the double knockout rescues both filamentation and biofilm formation, and even enhances these phenotypes under certain conditions, including biofilm formation in a mouse catheterization model. The authors go on to characterize many aspects of the knockout lines, including gene expression profiles and interactions with other loci previously shown to be important for these phenotypes. The properties described here are somewhat strain specific and the paper does not solve what is clearly an exceptionally complex regulatory hierarchy, nonetheless the manuscript provides new insights into the gene expression cascades that determine biofilm formation and adds to our understanding of the regulatory network of transcription factors controlling it.

Reviewer #2: This manuscript reports on new mechanistic insight into C. albicans biofilm formation, which is significant because biofilms commonly underlie the development of lethal systemic infections. The key result of this manuscript is the surprising discovery that combining two mutations (efg1 and ndt80) that individually cause defects in biofilm formation results in the opposite phenotype – biofilm formation that is better than either single mutant alone. This surprising genetic effect was most obvious in the SC5314 strain background, but the efg1 ndt80 combination resulted in improved hyphal growth in 5 other clinical isolates, strengthening the significance of this genetic interaction. Further genetic analysis showed that biofilm formation in the efg1 ngt1 strain was regulated by only a subset of the genes that have been identified previously to contribute to biofilm formation (BCR1, UME6, and HGC1), which correlated with the increased expression of these genes in the efg1 ndt80 strain. Interestingly, comparison of RNAseq data revealed that one of the commonly regulated genes was FLO9, a putative adhesin. Deletion of FLO9 essentially blocked biofilm in the efg1 ndt80 strain, highlighting a key role in this strain background. Altogether, these data show that a “stripped down” version of the complex biofilm circuitry is sufficient to drive biofilm formation, which now provides a new avenue for identifying regulators of this complex process.

Reviewer #3: The study by Do et al. reports the unexpected finding that the efg1Δ/Δ ndt80Δ/Δ double mutant of Candida albicans, despite the severe biofilm defects of each single mutant, is capable of forming robust biofilms in both in vitro and in vivo models. The authors define this as an “emergent biofilm” phenotype and provide evidence that it is mechanistically distinct from the conventional biofilm regulatory program. Through transcriptomic and genetic analyses, the study identifies key roles for Hgc1, Bcr1, Ume6, and the adhesin Flo9 in supporting this alternative biofilm state, while demonstrating independence from several canonical biofilm regulators, including Brg1, Rob1, Tec1, and Wor3. Overall, the work reveals an unexpected plasticity in the C. albicans biofilm regulatory network and suggests the existence of a simplified or primordial surface colonization program. The findings are interesting and provide new insight into how fungal virulence regulatory networks can adapt and reorganize.

**********

**Part II – Major Issues: Key Experiments Required for Acceptance**

Please use this section to detail the key new experiments or modifications of existing experiments that should be absolutely required to validate study conclusions.

Generally, there should be no more than 3 such required experiments or major modifications for a "Major Revision" recommendation. If more than 3 experiments are necessary to validate the study conclusions, then you are encouraged to recommend "Reject".

Reviewer #1: None

Reviewer #2: 1. It is interesting that the authors refer to the efg1 ndt80 genetic interaction as “emergent biofilm” formation. However, for clarity the authors should also state that it is a variation on the “synthetic” genetic interactions that have been widely used to study positive and negative interactions between genes.

2. The SC5314 strain differs from other clinical isolates in that it the two different alleles of ROB1 that differ in how they promote filament formation. I think it would be interesting for the authors to comment on the fact that deletion of ROB1 did not impact the efg1 ndt80 phenotype, indicating that the ROB1 alleles were not the critical difference for the SC5314 strain phenotype caused by efg1 ndt80.

3. FLO9 was described as a “putative adhesin”. The authors should supply more information about the basis for this description. Perhaps a supplemental figure showing the structural features on which this assessment is based? Is FLO9 conserved in other biofilm-forming species?

4. The NCBI SRA accession number PRJNA1453028 did not work. Please check it is correct.

Reviewer #3: Major comments:

1. Is Flo9 overexpression sufficient to drive biofilm formation in the efg1 or ndt80 single mutants?

2. Does the deletion of the Ume6 interacting protein, Upc2, affect the emergent biofilm formation? That would tell whether new binding partners are required.

**********

**Part III – Minor Issues: Editorial and Data Presentation Modifications**

Please use this section for editorial suggestions as well as relatively minor modifications of existing data that would enhance clarity.

Reviewer #1: Overall, the manuscript is thorough, and the data presented in a clear and easily understood manner. I have only minor comments for the authors to consider.

1. In the paragraph beginning on line 87, the authors mention that both efg1 and ndt80 knockout lines display defects in biofilm formation and filamentation, leading the reader to assume that they phenocopy each other. However, from the last sentence of this paragraph, it is clear that the two mutants share some characteristics and differ in others. For unfamiliar readers, it would be helpful to know how similar these mutant lines are. If there are additional data from previous characterizations of these single mutant lines, it would be great if the authors could provide a brief summary.

2. In the section beginning on line 163, the authors compare RNA-seq data sets from a wild type line and the efg1/ndt80 double mutant. This is, of course, both interesting and key to the analysis that the authors are presenting. However, it would also be interesting to know how the double mutant compares to each single mutant (and perhaps how the single mutants compare to each other). Ideally, the RNA-seq analyses of all these lines would be performed in parallel, however this might be prohibitive at this point. Are there preexisting RNA-seq datasets for each of the single mutant lines that could be used for comparison, even if the comparison is qualitative rather than quantitative (e.g. are similar/different pathways up/down regulated)?

Reviewer #2: • Lines 63-65. Sentence unclear – missing a word?

• Line 355 to around Line 390 – define YFG1 and YFG

• Lines 411-412. Unclear sentence. What is OD0.5?

• define ddH2O

Reviewer #3: Minor comments:

1. It would be good to also include the phenotype of the single mutants in Fig. 4A, 4B, and 4C.

2. In Fig. 5, please add what RNA-seq conditions from other studies were used to generate 5B.

3. It would be good to add to the discussion why the emergent biofilm of the double mutant is only in SC5314, but not in other strain background. In addition, it would be good to discuss what makes Flo9 so special to be required for emergent biofilm formation, but not other adhesins. Is the regulation of Flo9 much different compared to other adhesins?

4. Line 64: add “condensates” after “phase-separated”

**********

PLOS authors have the option to publish the peer review history of their article (what does this mean?). If published, this will include your full peer review and any attached files.

If you choose “no”, your identity will remain anonymous but your review may still be made public.

**Do you want your identity to be public for this peer review?**  For information about this choice, including consent withdrawal, please see our Privacy Policy.

Reviewer #1: No

Reviewer #2: No

Reviewer #3: No

[NOTE: If reviewer comments were submitted as an attachment file, they will be attached to this email and accessible via the submission site. Please log into your account, locate the manuscript record, and check for the action link "View Attachments". If this link does not appear, there are no attachment files.]

**Figure resubmission:**

-->While revising your submission, we strongly recommend that you use PLOS’s NAAS tool (https://ngplosjournals.pagemajik.ai/artanalysis) to test your figure files. NAAS can convert your figure files to the TIFF file type and meet basic requirements (such as print size, resolution), or provide you with a report on issues that do not meet our requirements and that NAAS cannot fix.

After uploading your figures to PLOS’s NAAS tool - https://ngplosjournals.pagemajik.ai/artanalysis, NAAS will process the files provided and display the results in the "Uploaded Files" section of the page as the processing is complete. If the uploaded figures meet our requirements (or NAAS is able to fix the files to meet our requirements), the figure will be marked as "fixed" above. If NAAS is unable to fix the files, a red "failed" label will appear above. When NAAS has confirmed that the figure files meet our requirements, please download the file via the download option, and include these NAAS processed figure files when submitting your revised manuscript.

**Reproducibility:**

To enhance the reproducibility of your results, we recommend that authors of applicable studies deposit laboratory protocols in protocols.io, where a protocol can be assigned its own identifier (DOI) such that it can be cited independently in the future. Additionally, PLOS ONE offers an option to publish peer-reviewed clinical study protocols. Read more information on sharing protocols at https://plos.org/protocols?utm_medium=editorial-email&utm_source=authorletters&utm_campaign=protocols

# Author comment: Author response to Decision Letter 1

**DOI:** 10.1371/journal.ppat.1014469.r002

16 Jun 2026

Attachment Submitted filename: Response to Reviewers.docx

# Peer review: Decision Letter 1

**DOI:** 10.1371/journal.ppat.1014469.r003

**Authors:** Michal Olszewski, Biao Ren

12 Jul 2026

Dear Professor Mitchell,

We are pleased to inform you that your manuscript 'An emergent biofilm program from inactivation of Candida albicans master regulators Efg1 and Ndt80' has been provisionally accepted for publication in PLOS Pathogens.

Before your manuscript can be formally accepted you will need to complete some formatting changes, which you will receive in a follow up email. A member of our team will be in touch with a set of requests.

Please note that your manuscript will not be scheduled for publication until you have made the required changes, so a swift response is appreciated.

IMPORTANT: The editorial review process is now complete. PLOS will only permit corrections to spelling, formatting or significant scientific errors from this point onwards. Requests for major changes, or any which affect the scientific understanding of your work, will cause delays to the publication date of your manuscript.

Should you, your institution’s press office or the journal office choose to press release your paper, you will automatically be opted out of early publication. We ask that you notify us now if you or your institution is planning to press release the article. All press must be co-ordinated with PLOS.

Thank you again for supporting Open Access publishing; we are looking forward to publishing your work in PLOS Pathogens.

Best regards,

Biao Ren

Academic Editor

PLOS Pathogens

Michal Olszewski

Section Editor

PLOS Pathogens

Sumita Bhaduri-McIntosh

Editor-in-Chief

PLOS Pathogens

orcid.org/0000-0003-2946-9497

Michael Malim

Editor-in-Chief

PLOS Pathogens

orcid.org/0000-0002-7699-2064

***********************************************************

Reviewer Comments (if any, and for reference):

Reviewer’s Responses to Questions

**Part I - Summary**

Please use this section to discuss strengths/weaknesses of study, novelty/significance, general execution and scholarship.

Reviewer #1: The manuscript provides intriguing and important information regarding the mechanisms regulating biofilm formation in the fungal pathogen Candida albicans. The authors employed a genetic approach to identify key determinants of this virulence associated property. The information included in the manuscript solidly supports the conclusions of the authors.

Reviewer #2: The authors have addressed my comments in a satisfactory manner.

Reviewer #3: The authors had a lovely first submission and have fully addressed any concerns.

**********

**Part II – Major Issues: Key Experiments Required for Acceptance**

Please use this section to detail the key new experiments or modifications of existing experiments that should be absolutely required to validate study conclusions.

Generally, there should be no more than 3 such required experiments or major modifications for a "Major Revision" recommendation. If more than 3 experiments are necessary to validate the study conclusions, then you are encouraged to recommend "Reject".

Reviewer #1: The authors have suitably addressed my previous comments. I have no further suggestions.

Reviewer #2: (No Response)

Reviewer #3: (No Response)

**********

**Part III – Minor Issues: Editorial and Data Presentation Modifications**

Please use this section for editorial suggestions as well as relatively minor modifications of existing data that would enhance clarity.

Reviewer #1: The authors have suitably addressed my previous comments. I have no further suggestions.

Reviewer #2: (No Response)

Reviewer #3: (No Response)

**********

PLOS authors have the option to publish the peer review history of their article (what does this mean?). If published, this will include your full peer review and any attached files.

If you choose “no”, your identity will remain anonymous but your review may still be made public.

**Do you want your identity to be public for this peer review?**  For information about this choice, including consent withdrawal, please see our Privacy Policy.

Reviewer #1: No

Reviewer #2: No

Reviewer #3: No

# Editor report: Acceptance letter

**DOI:** 10.1371/journal.ppat.1014469.r004

**Authors:** Michal Olszewski, Biao Ren

Dear Professor Mitchell,

We are delighted to inform you that your manuscript, "An emergent biofilm program from inactivation of Candida albicans master regulators Efg1 and Ndt80," has been formally accepted for publication in PLOS Pathogens.

We have now passed your article onto the PLOS Production Department who will complete the rest of the pre-publication process. All authors will receive a confirmation email upon publication.

The corresponding author will soon be receiving a typeset proof for review, to ensure errors have not been introduced during production. Please review the PDF proof of your manuscript carefully, as this is the last chance to correct any scientific or type-setting errors. Please note that major changes, or those which affect the scientific understanding of the work, will likely cause delays to the publication date of your manuscript. Note: Proofs for Front Matter articles (Pearls, Reviews, Opinions, etc...) are generated on a different schedule and may not be made available as quickly.

Soon after your final files are uploaded, the early version of your manuscript, if you opted to have an early version of your article, will be published online. The date of the early version will be your article’s publication date. The final article will be published to the same URL, and all versions of the paper will be accessible to readers.

For Research Articles, you will receive an invoice from PLOS for your publication fee after your manuscript has reached the completed accept phase. If you receive an email requesting payment before acceptance or for any other service, this may be a phishing scheme. Learn how to identify phishing emails and protect your accounts at https://explore.plos.org/phishing.

Thank you again for supporting open-access publishing; we are looking forward to publishing your work in PLOS Pathogens.

Best regards,

Sumita Bhaduri-McIntosh

Editor-in-Chief

PLOS Pathogens

orcid.org/0000-0003-2946-9497

Michael Malim

Editor-in-Chief

PLOS Pathogens

orcid.org/0000-0002-7699-2064
